# Advances in Fluorescence Techniques for the Detection of Hydroxyl Radicals near DNA and Within Organelles and Membranes

**DOI:** 10.3390/antiox14010079

**Published:** 2025-01-10

**Authors:** Eleanor C. Ransdell-Green, Janina Baranowska-Kortylewicz, Dong Wang

**Affiliations:** Department of Pharmaceutical Sciences, College of Pharmacy, University of Nebraska Medical Center, Omaha, NE 68198, USA; eransdellgreen@unmc.edu

**Keywords:** hydroxyl radicals, reactive oxygen species, fluorescence detection, DNA-targeting, organelle-targeting, coumarin-based probes, oxidative stress

## Abstract

Hydroxyl radicals (^•^OH), the most potent oxidants among reactive oxygen species (ROS), are a major contributor to oxidative damage of biomacromolecules, including DNA, lipids, and proteins. The overproduction of ^•^OH is implicated in the pathogenesis of numerous diseases such as cancer, neurodegenerative disorders, and some cardiovascular pathologies. Given the localized nature of ^•^OH-induced damage, detecting ^•^OH, specifically near DNA and within organelles, is crucial for understanding their pathological roles. The major challenge of ^•^OH detection results from their short half-life, high reactivity, and low concentrations within biological systems. As a result, there is a growing need for the development of highly sensitive and selective probes that can detect ^•^OH in specific cellular regions. This review focuses on the advances in fluorescence probes designed to detect ^•^OH near DNA and within cellular organelles and membranes. The key designs of the probes are highlighted, with emphasis on their strengths, applications, and limitations. Recommendations for future research directions are given to further enhance probe development and characterization.

## 1. Introduction

Intracellular reactive oxygen species (ROS) are a diverse class of highly reactive molecules that consist of both free radicals and certain non-radical oxygen agents capable of forming free radicals. Examples of free radicals include the superoxide anion radical (O_2_^•−^), hydroxyl radical (^•^OH), peroxyl radical (RO_2_^•^), and alkoxyl radical (RO^•^). Non-radicals include hydrogen peroxide (H_2_O_2_), singlet oxygen (^1^O_2_), and hypochlorous acid (HOCl) [1]. These molecules are produced both as natural byproducts of cellular metabolism and as deliberate products of enzymatic reactions. A primary source of ROS in cells is the electron transport chain (ETC) in mitochondria, where the partial reduction of oxygen during aerobic respiration generates superoxide radicals [2,3,4]. ROS are also generated within the endoplasmic reticulum (ER) [5] and can be formed in enzymatic reactions involving NADPH oxidases (NOX) and other oxidases [6]. Through their involvement in redox reactions, ROS function as secondary messengers in signaling cascades, influencing cell growth, differentiation, and apoptosis by modulating the activity of transcription factors and enzymes [7]. ROS are also involved in various physiological functions, such as muscle contractions and vascular tone regulation [8,9,10,11]. In the immune system, ROS help phagocytes like neutrophils and macrophages in the destruction of pathogens [12]. Consequently, ROS are a vital component in many cellular functions and have significant biological importance.

While ROS serve important cellular functions at regulated levels, excessive production can lead to oxidative stress. At uncontrolled levels, ROS can quickly overwhelm the cell’s natural antioxidant defense systems and react non-specifically with cellular materials like proteins, lipids, and nucleic acids. When these reactions occur, they can initiate chain reactions that cause significant changes in the structure and function of essential biomolecules [13], for example, causing oxidative damage-induced DNA mutations [14], lipid peroxidation [15], and protein oxidation [16]. These changes can compromise cellular functions and exacerbate the consequences of oxidative stress. ROS-induced oxidative stress has been implicated in diverse human pathologies, such as ischemia–reperfusion injury, hypertension, inflammation, cystic fibrosis, type-2 diabetes, cardiovascular diseases, atherosclerosis, cancer, and neurodegenerative disorders [8,17,18]. ROS production can be induced by exogenous sources such as alcohol, tobacco smoke, pollution, drugs, and ionizing radiation, which can lead to irreversible effects on tissue development [19,20,21].

Among the intracellular ROS, the hydroxyl free radical (^•^OH) is of particular concern due to its high reactivity. The hydroxyl radical possesses an unpaired electron in the outermost shell of the oxygen atom, giving it an exceptionally potent one-electron reduction potential of 2.31 V [22,23]. Within biological systems, ^•^OH have a fleeting half-life of approximately 10^−9^ s and engage in near-diffusion-limited reactions with all known organic molecules, exhibiting rate constants ranging from 10^9^ to 10^10^ M^−1^ s^−1^ [24]. With a mean diffusion distance of ~6 nm [25], the ^•^OH interacts with its targets through processes like hydrogen abstraction and/or addition and the electron transfer mechanism [26]. Their indiscriminate interactions with co-reactants can lead to widespread cellular damage by targeting virtually all biological molecules within their immediate vicinity. This oxidative damage disrupts cellular processes and positions ^•^OH overproduction as a key driver of oxidative stress.

Detecting intracellular ^•^OH variations can offer valuable insights into the intricate dynamics of redox regulation and the pathological effects of its imbalance. However, their high reactivity and short lifespan result in low concentrations of ^•^OH in biological systems, which presents significant challenges for detecting and studying their role in pathological diseases. Therefore, the development of highly sensitive and selective detection methods for monitoring ^•^OH is of high importance. Many techniques have been developed for the detection and quantitative determination of ^•^OH, including electron spin resonance (ESR) spectroscopy [27,28], UV-vis spectrophotometry [29,30], electrochemical sensing [31,32], chromatography [33], chemiluminescence [34,35], and fluorescence [36,37]. Among these methods, fluorescent probes have shown great promise. Fluorescence detection offers high selectivity and sensitivity, as well as other advantages such as rapid response rate, non-invasive imaging, superior spatio-temporal resolution, and low cost. Furthermore, fluorescence analysis is highly effective for real-time detection and high-resolution cell imaging, which will provide detailed information on ^•^OH variations at specific intracellular locations [38].

Several review articles focus on the development of fluorescence techniques for the selective monitoring of ^•^OH under biological conditions [38,39,40,41]. Alanazi et al. (2024) provide a thorough discussion of the recent advances in ^•^OH-responsive fluorescent nanoprobes [39], and Hou et al. (2020) offer a detailed review of fluorescent ^•^OH probes specifically for bioimaging applications [40]. However, despite advancements in fluorescence techniques for ^•^OH detection, there are notably few reviews specifically focused on ^•^OH-responsive fluorescent sensors that target specific intracellular sites. This review examines the properties and formation of ^•^OH, their roles and impacts within cells, and the advancements made in developing fluorescent probes designed to detect and measure ^•^OH at specific cellular locations, including those in the vicinity of DNA and those within organelles and membranes. Highlighted are the probe’s key designs, along with their strengths, applications, and limitations. Additionally, recommendations for future research directions will be provided to refine experimental strategies.

### 1.1. Mechanisms of Hydroxyl Radical Formation

**Figure 1:**
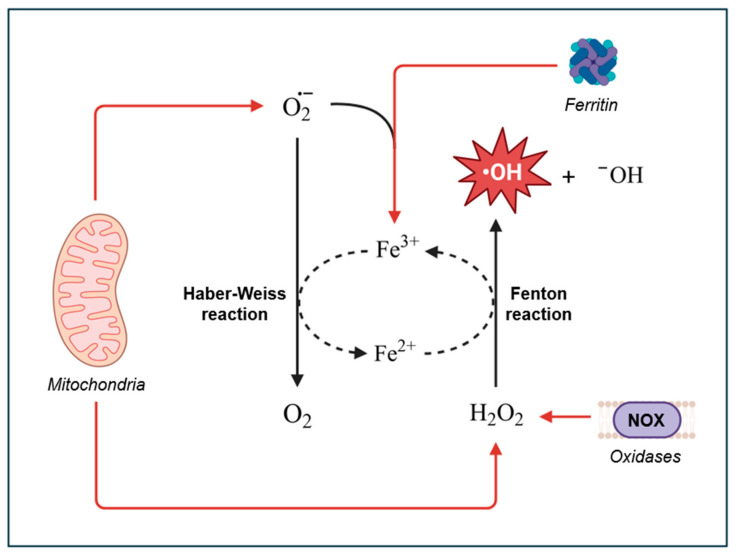
The Fenton/Haber–Weiss reaction. Superoxide radicals produced from the mitochondria can react with ferritin complexes, releasing ferric ions. These unbound ferric ions can be reduced in the Haber–Weiss reaction to produce ferrous ions. Ferrous ions catalyze the Fenton reaction to generate ^•^OH from H_2_O_2_ produced by mitochondria and NOX oxidases. The ferric ions produced from the Fenton reaction can then be recycled by reduction through superoxide radicals, resulting in continuous ^•^OH production.

Hydroxyl radicals can be generated in vivo through several pathways, with the Fenton reaction being the primary mechanism. In the Fenton reaction, hydrogen peroxide (H_2_O_2_) reacts with ferrous ion (Fe^2+^) or other transition metals to produce ^•^OH [42]. In the body, these metal ions are sequestered by proteins like ferritin or ceruloplasmin to prevent uncontrolled reactions [43,44,45,46]. However, during periods of oxidative stress, the body can generate excess superoxide radicals, which can release the metal ions from their protein complexes. Once unbound, these metals are free to participate in Fenton reactions, generating highly reactive ^•^OH [47,48,49]. Notably, the ferric ions produced from the Fenton reaction can be reduced back into ferrous ions by superoxide radicals. This reduction, combined with the Fenton reaction, results in the net reaction known as the Haber–Weiss reaction (Figure 1).

This mechanism of ^•^OH generation is particularly relevant to conditions like ferroptosis, in which labile iron-dependent ROS production leads to lipid peroxidation and subsequent regulated cell death [50,51].

Another mechanism which generates ^•^OH is effectuated through the radiolysis of water by ionizing radiation [52]. When exposed to ionizing radiation (IR), water is converted to a water cation radical (H_2_O^•+^) (Equation (2)). Subsequently, a proton is rapidly transferred from H_2_O^•+^ to a nearby water molecule, resulting in the formation of ^•^OH (Equation (1)).

(1) $$H_{2}O\overset{Ionizing\;radiation}{\to }H_{2}O^{•+}+e_{aq}^{-}$$

(2) H2O•++H2O→H3O++OH•

Additionally, ^•^OH can be generated through molecular fragmentation of the excited state of water (Equation (3)). In this process, the energy from IR breaks the bonds of the water molecules, which leads to the formation of ^•^OH and hydrogen atoms [52].

(3) H2O→Ionizing radiationH2O*→H•+OH•

These mechanisms demonstrate the central role of ^•^OH in mediating the indirect effects of radiation-induced damage to DNA and other biomacromolecules.

### 1.2. Major Intracellular Sources of ^•^OH

Hydroxyl radicals and other ROS are generated at several different cellular locations. The main source of intracellular ROS is the electron transport chain (ETC) within the inner mitochondrial membrane and cellular sites containing NADPH oxidases. These major ROS sources are tightly regulated by the cell’s antioxidant defense systems. However, when these defenses are overwhelmed, excessive ROS can accumulate.

#### 1.2.1. The Mitochondrial Electron Transport Chain

Mitochondria are the primary source of ROS generation through the ETC. Although the primary role of the ETC is to facilitate the reduction of oxygen to water by cytochrome oxidase (Complex IV), the alternating one-electron transfers within the chain can lead to side reactions and electron leakage. The two main sites of electron leakage are complex I and complex III, where electrons flowing from reduced substrates can be inadvertently transferred to molecular oxygen, forming unstable superoxide anions [53]. Iron–sulfur clusters in the ETC can also contribute to the conversion of oxygen into superoxide [2]. The superoxide anions are then converted to H_2_O_2_ by superoxide dismutase (SOD), which can readily diffuse through the membrane and participate in Fenton reactions to generate ^•^OH. To prevent excessive production of ^•^OH, the cell utilizes peroxide scavenging (peroxidase) systems to tightly control H_2_O_2_ levels by reducing H_2_O_2_ to H_2_O [54]. These systems, along with other non-enzymatic antioxidants (e.g., vitamins), work to maintain a healthy balance between ROS production and elimination [55].

In some instances, ROS production can overwhelm the cell’s antioxidant systems of ROS defense. One significant factor contributing to this imbalance is mitochondrial calcium (Ca^2+^) overload. Under normal physiological conditions, Ca^2+^ serves to enhance mitochondrial function by stimulating oxidation-phosphorylation and ATP synthesis [56]. However, when calcium levels become dysregulated, an accumulation of Ca^2+^ can trigger the opening of the mitochondrial permeability transition pore (mPTP) [57]. The opening of the mPTP disrupts the mitochondrial membrane potential and impairs ETC function, resulting in increased electron leakage and ROS production. Ultimately, this can lead to cell death through necrosis or apoptosis [58]. Mitochondrial Ca^2+^ overload has been implicated in neurodegenerative diseases like amyotrophic lateral sclerosis (ALS) and Alzheimer’s disease [57,59,60], as well as heart failure [61].

#### 1.2.2. NADPH Oxidases

The primary sources of ROS in cells, besides the ETC, are NADPH oxidases (NOX). NOX are unique in that their primary function is to produce ROS. Hence, they are often referred to as “professional” ROS producers. NOX can be found within the plasma membrane, endoplasmic reticulum, mitochondrial membrane, nuclear membrane, and specialized microdomains such as caveoli and lipid rafts, as well as focal adhesions and invadopodia [62,63,64,65,66,67]. As enzymes, NOX catalyze the production of O_2_^•−^ by the transferring of electrons from NADPH to oxygen (Equation (4)). As mentioned above, O_2_^•−^ can be converted to H_2_O_2_ by SOD, which can then participate in Fenton reactions to generate ^•^OH.

(4) $$NADPH+2O_{2}^{•-}\overset{NADPH\;oxidase}{\to }2O_{2}+{NADP}^{+}+H^{+}$$

NOX enzymes exist in several different isoforms, which are classified based on their tissue distribution and mechanism of action. All isoforms ultimately contribute to the production of ^•^OH through their generation of superoxide radicals (NOX1, NOX2, and NOX5) and H_2_O_2_ (NOX4) [68].

NOX-derived ROS are essential for cellular signaling within the vascular system, but proper regulation of NOX activity is crucial. Both insufficient and excessive levels of NOX-generated ROS can disrupt vascular health. In particular, the overexpression of NOX is implicated in increased oxidative stress, which in turn accelerates vascular damage and promotes the progression of diseases related to oxidative injury [69,70,71].

## 2. Essential Criteria for Effective Fluorescent Probes in ^•^OH Detection

To reliably detect and measure ^•^OH in biological settings, fluorescent probes must meet several key criteria (Table 1). Firstly, it is important that the probe exhibits high selectivity and specificity for ^•^OH, with minimal interference from other common ROS. Because intracellular ^•^OH are present at low concentrations, the probe must also offer a high degree of sensitivity for ^•^OH. This is usually reflected by a high fluorescence quantum yield (QY). Fluorescence QY refers to the fraction of excited molecules that return to the ground state by emitting a fluorescence photon. Thus, it provides a direct measure of the efficiency of the fluorescence process [72]. Other important criteria include high photostability, stability across a relevant pH range, and resistance to interference from factors such as radiation, transition metals (two common sources of ^•^OH generation), and other cellular components like proteins and ions. Carrying out in vitro tests under biologically relevant conditions will ensure that the probe remains effective for reliable detection within cells with complex biological systems.

When designing probes to target specific cellular sites, like those near DNA or within organelles, it is crucial that the probes effectively bind to or accumulate at the intended site. This can be achieved by adding one or more targeting moieties. The binding of the probe to macromolecules, such as histones or DNA, must not significantly alter the structure of the macromolecule or interfere with the probe’s performance. For cell culture applications, the probe should exhibit good water solubility and cell permeability to ensure quick uptake and distribution within subcellular compartments. Furthermore, the probe must be non-toxic and have high biocompatibility at the probe concentrations needed for a strong and reliable fluorescence signal.

## 3. Fluorescence Detection of DNA-Associated ^•^OH

Since the 1950s, ^•^OH have been recognized for their ability to damage DNA, primarily through their oxidation of constituent bases. This damage can occur through the addition of ^•^OH to the double bonds of DNA bases, or by the site-specific abstraction of hydrogen atoms. These interactions can result in the formation of radical adducts and oxidative lesions [73]. The primary classes of ^•^OH-mediated oxidative damage include base damage or base loss [74], DNA strand breaks [75], thymine dimers [76], DNA-protein [77], DNA-DNA cross-links [78,79], and DNA-DNA intrastrand adducts [80,81]. Such damage can have profound consequences on DNA structure and can potentially alter DNA replication, causing genetic rearrangements, mutations, and DNA strand breaks [82]. Double-strand breaks (DSB) in DNA can result in apoptosis [83], directly inactivate key genes, or induce chromosomal aberrations [84,85]. Additionally, ^•^OH-induced DNA damage has been implicated in numerous diseases including cancer [86], neurodegenerative disorders such as Alzheimer’s disease and Parkinson’s disease [87,88], cardiovascular diseases [89], diabetes [90], and various inflammatory conditions [91]. Thus, it is vital to improve our understanding of the dynamics of ^•^OH-induced DNA damage and how it contributes to disease progression. The short-lived nature of ^•^OH and the complexity of cellular environments necessitate the development of more sensitive and targeted detection methods for DNA-associated ^•^OH.

### 3.1. Coumarin-Based Probes

**Figure 2:**
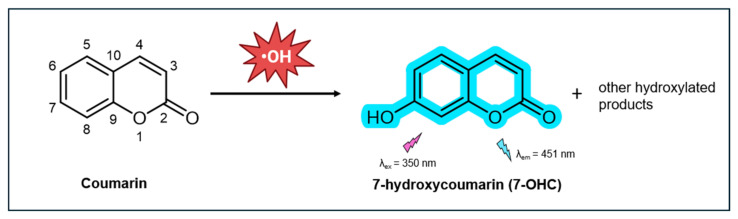
The hydroxylation of coumarin by ^•^OH to produce the fluorescent 7-hydroxycoumarin, along with other hydroxylated products. The coumarin framework is numbered according to the IUPAC numbering system.

Coumarin-based probes have emerged as powerful tools for detecting and quantifying ^•^OH due to their distinctive fluorescence properties [92,93,94,95]. Due to its aromatic structure, coumarin is highly reactive with ^•^OH, which tend to attack the most electron-rich sites on the ring. In neutral aqueous solutions, this reaction results in hydroxylation at the 7-position, resulting in the formation of 7-hydroxycoumarin (7-OHC) (Figure 2). Although other oxidation products are formed, 7-OHC is notably the only product that exhibits strong fluorescence, with a high QY of 0.5 at normal physiological conditions [96]. This makes it highly suitable for sensitive detection of ^•^OH. Coumarin and its derivatives are particularly valued as fluorescent tools for their large Stokes shift, high quantum yield, biocompatibility, cell permeability, and straightforward synthesis and modifications [97,98]. Coumarin is readily available as a commercial probe for ^•^OH detection, making it accessible for widespread use in research.

**Figure 3:**
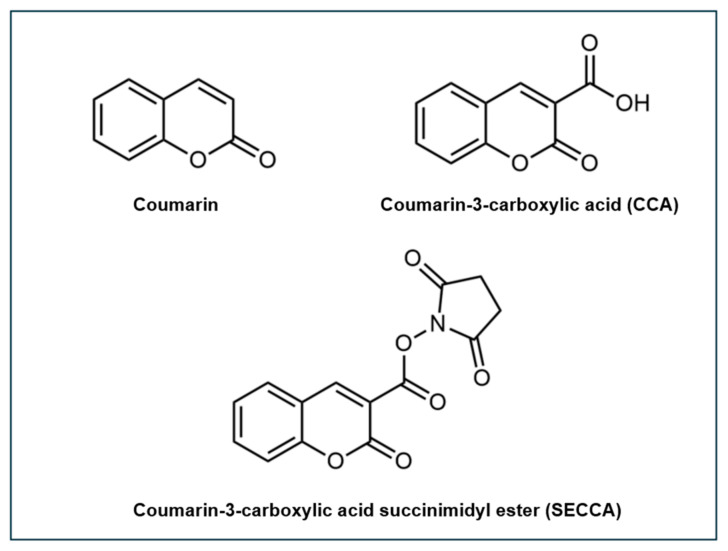
Chemical structures of coumarin and its derivatives, which are commonly used for hydroxyl radical detection.

One notable derivative of coumarin, another commercially available probe, is coumarin-3-carboxylic acid (CCA) (Figure 3). Unlike coumarin, CCA features, at the C3 position, a carboxylic group, preventing hydroxylation at this position. Moreover, the presence of the carboxylic group enables the coupling of CCA to the amine groups of other molecules such as amine-functionalized nanoparticles [93,99] and phospholipids [100].

Building upon the properties of CCA, coumarin-3-carboxylic acid succinimidyl ester (SECCA) has been developed to enhance specificity for DNA-related applications. SECCA is a further derivatized form of CCA, one which can be more effectively conjugated to primary amines in biomolecules such as polypeptides, proteins, and nucleic acids (Figure 3). When SECCA, a non-fluorescent compound, interacts with ^•^OH, it is converted into hydroxylated products, including the highly fluorescent 7-hydroxy-SECCA (7-OH-SECCA). This fluorescence has been proven to be specifically induced by ^•^OH and remains unresponsive to other ROS [101,102]. By conjugating SECCA to DNA and utilizing its specificity for ^•^OH, the induced fluorescence offers a quantitative measure of ^•^OH formation in the immediate vicinity of DNA, minimizing background fluorescence from the solution. This enables us to derive information that reflects ^•^OH attack and the extent of ^•^OH-induced damage upon the biomolecule itself. Many studies have focused on the development and application of SECCA-based probes for detecting ^•^OH in DNA contexts. SECCA has been utilized in the conjugation to the lysines of polylysine-DNA and histone-H1-DNA complexes [101,102,103,104,105,106].

### 3.2. SECCA-Biomolecule Conjugation

Makrigiorgos et al. pioneered a method to quantify radiation-induced ^•^OH near DNA by conjugating it with SECCA [101]. Their technique enabled the attachment of one to five SECCA molecules to biomolecules such as polylysine, human serum albumin (HSA), avidin, histone-H1, and amino-functionalized oligonucleotides. This approach proved highly sensitive, allowing the detection of fluorescent signals from radiation doses as low as 0.01 Gy. Through a series of control studies, they demonstrated that the fluorescence induced by SECCA is specifically mediated by ^•^OH, and that the presence of oxygen enhances this fluorescence by a factor of 4. Additionally, it was shown that the induced fluorescence of SECCA has a direct relationship with the radiation dose and is not significantly influenced by other primary and secondary water radicals. This preliminary study laid the groundwork for subsequent developments, leading to the application of SECCA-labeled polylysine and histone-H1 for complexation with DNA, further advancing the ability to study ^•^OH-induced oxidative damage in genetic material.

#### 3.2.1. SECCA-Labeled Histone-H1

The technique of SECCA–biomolecule conjugates has been applied to chromatin modeling systems through the complexation of SECCA-labeled histone-H1 to DNA [102,104,105]. Upon the conjugation of SECCA to histone-H1, it was found that the chemical modification of the histone at a low average molar ratio of SECCA/histone (~two SECCA/histone) has no impact on its DNA binding properties or the conformation of the histone-DNA complex. Upon binding to DNA, the SECCA-histone-H1 complex showed a modest reduction of about 30% in quantum efficiency (QE). This reduction in QE was more pronounced when the molar ratio of SECCA to histone exceeded the above-mentioned level, and may be attributable to fluorescence energy transfer. Furthermore, the ability of SECCA–histone-H1 conjugates to access ^•^OH was reduced seven-fold when associated with nucleohistones. This is likely the result of the scavenging of ^•^OH by DNA and the decreased collision frequency of ^•^OH with the histone after its association with the DNA macromolecules. The authors estimated that free radical scavenging by DNA and decreased collision frequency contribute separate reduction factors of about 3.5 and 1.9, respectively [102].

Due to the random conjugation of SECCA to histone-H1, the generated signal is considered a random sampling which quantifies the ability of ^•^OH to access the whole population of histone H1. Because of the high reactivity of ^•^OH, the fluorescence signal should be proportional to the overall attack of ^•^OH on the biomolecule, with negligible ^•^OH shielding effects present due to the low degree of histone labeling with SECCA. Furthermore, it is assumed that, since the size of SECCA is ~4 Å, the portion of ^•^OH that reacts with SECCA is virtually within the histone [102].

#### 3.2.2. SECCA-Labeled Polylysines

SECCA-labeled polylysine-DNA complexes have also been explored for site-specific investigation of ^•^OH production near DNA. In their 1993 study, Makrigiorgos et al. described a protocol enabling the covalent attachment of five SECCA molecules per polylysine [101], while subsequent studies utilized a molar ratio of 1–2 SECCA labels per polylysine [105,106]. The SECCA–polylysine conjugates are water soluble, with little to no reduction in the QE of 7-OH-SECCA upon binding to polylysine [101,105]. A slight reduction in fluorescence induction of SECCA-polylysine relative to free SECCA indicates that polylysine competes with SECCA in the scavenging of ^•^OH [101]. These SECCA–polylysine conjugates can be effectively complexed with DNA using low salt conditions [106].

#### 3.2.3. SECCA-Labeled Nucleosomal Histones

The histone H1/polylysine-DNA systems used in the initial research by Makrigiorgos et al. [102,105] do not accurately represent the chromatin structures found in cells. To improve upon this, Chakrabarti et al. developed a method to attach SECCA to the aliphatic amines of histones within nucleosomal core particles [107]. They showed that by using one SECCA molecule per nucleosomal histone, strong and proportional fluorescence signals could be achieved during exposure to gamma radiation or copper–ascorbic acid–hydrogen peroxide reactions. Importantly, electron microscopy and micrococcal nuclease digestion confirmed that the nucleosomal structure remained intact after labeling, indicating that there had been no disruption of the chromatin particles. The fluorescence induction of the probe was shown to be highly specific to ^•^OH, as the introduction of DMSO, an effective ^•^OH scavenger, efficiently quenched the signal. Overall, the SECCA probe has proved to be a highly effective tool for continuous and sensitive detection of chromatin-associated ^•^OH.

### 3.3. Applications of SECCA-Labeled Biomolecules

In a series of preliminary studies, SECCA-polylysine-DNA and Histone-H1-DNA complexes have been successfully used to detect and quantify ^•^OH generated by different sources, including radiation and transition metals as well as DNA-binding agents. These studies have demonstrated SECCA’s high sensitivity and specificity in these applications.

#### 3.3.1. Detection of Radiation-Induced ^•^OH

SECCA–biomolecule conjugates have demonstrated significant potential for detecting ^•^OH induced by radiation [101,102,104,105,107]. Kinetic studies have shown a direct relationship between the induced fluorescence of SECCA and SECCA-labeled biomolecules and the radiation dose rates [101,102,105,107]. Importantly, the direct action of radiation, whether from low- or high-LET sources, does not significantly affect the fluorescence induction [102]. Other radiolytically-produced species such as $e_{aq}^{-}$, H^•^, HO_2_^•^, and O_2_^•−^ do not notably contribute to the induced fluorescence. This is supported by a 1:1 stoichiometric relationship between ^•^OH and the fluorescence signal, indicating that the fluorescence from 7-OH-SECCA specifically reflects the presence of ^•^OH [101,102]. Once formed, the fluorescent 7-OH-SECCA conjugates are stable and do not undergo further changes due to radiation, maintaining their integrity for at least 7 days post-irradiation [101]. This stability is particularly advantageous for continuous monitoring when prolonged or low-dose radiation exposures, such as those with unsealed sources of radiation, are involved.

From the preliminary studies, Makrigiorgos et al. have demonstrated that SECCA-labeled histone-H1 can effectively detect and quantify ^•^OH within chromatin sites at doses ranging from 0.3 to 30 Gy [102]. Furthermore, because fluorescence intensity is not substantially altered by NaCl concentration (14–800 mmol dm^−3^), SECCA-labeled histone-H1 and SECCA-labeled polylysine were successfully applied in a fluorescence polarization spectroscopy study. This study investigated the relationship between the conformational state of nucleic-acid-complexed biomolecules in simple chromatin models and their accessibility to radiation-induced ^•^OH [105].

#### 3.3.2. Detection of Metal-Mediated ^•^OH

In investigations of metal-mediated hydroxyl radicals, the potential effects of nearby metal ions on SECCA fluorescence need to be considered. Metal cations, such as Fe^2+^, Fe^3+^, and Cu^2+^, quench fluorescence when the distance between the metal and fluorophore is less than a few tens of angstroms [108]. Thus far, reported data indicate that the fluorescence method using SECCA can be effectively applied to chromatin-associated ^•^OH production in the presence of metal ions [103,104,106,109]. For copper, it is recommended to avoid using exceedingly high concentrations (≥100 µmol dm^−^^3^) to prevent significant fluorescence quenching of 7-OH-SECCA. In addition, there is no evidence indicating that the presence of the detector molecule SECCA significantly impacts the distribution of copper or the generated ^•^OH [106]. It has been shown that singlet oxygen, potentially catalyzed by Cu, is unable to convert SECCA to 7-OH-SECCA [104].

Both SECCA-labeled histone-H1 and SECCA-labeled polylysine DNA complexes have been used for the detection of ^•^OH generated by metal-bound DNA-binding agents in the presence of ascorbate and H_2_O_2_ [103,104]. In 1996, Chakrabarti et al. successfully applied SECCA-polylysine-DNA complexes to demonstrate the generation of ^•^OH by Fe(II)-bleomycin in the vicinity of DNA [103]. In another 1996 study by Chakrabarti et al., both SECCA-polylysine-DNA complexes and SECCA-histone-H1-DNA complexes were used to demonstrate the generation of chromatin-associated ^•^OH by nucleohistone-bound metal ion-Adriamycin [104]. Together, these two studies illustrate the utility of SECCA–biomolecule conjugates in revealing DNA-associated ^•^OH as a factor contributing to DNA damage induced by metal ion-bound DNA-binding bleomycin and Adriamycin, two commonly used chemotherapeutic drugs. Significantly, both studies revealed that intracellular scavengers may be ineffective in preventing ^•^OH from attacking chromatin [103,104].

### 3.4. Other DNA-Targeting Coumarin-Based Probes in Development

**Figure 4:**
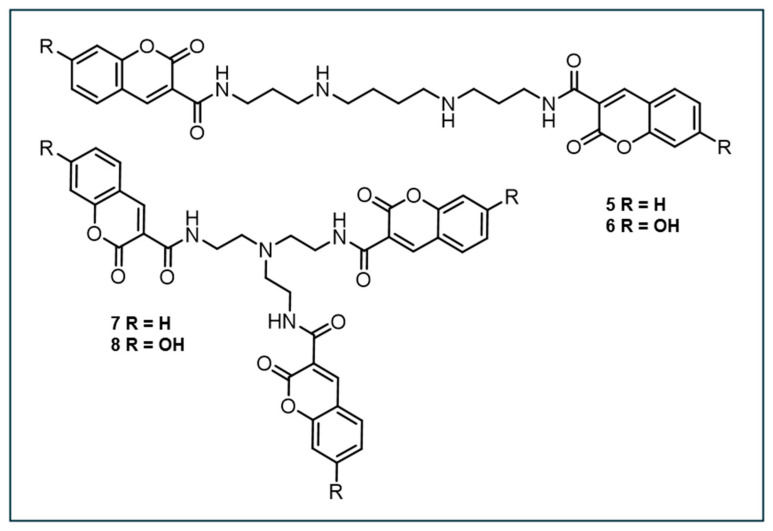
Chemical structures of coumarin–polyamine conjugates **5**–**8**.

In 2007, Singh et al. designed four low-molecular coumarin–polyamine-derived conjugates (compounds **5**–**8**, Figure 4) to detect ^•^OH near DNA. These probes incorporate a polyamine moiety to bind DNA without affecting its structure [110]. Prior to synthesis, molecular docking showed that the curvature of coumarin analogs fits into the DNA minor groove and is stabilized by hydrogen bonds. This enabled them to determine the binding free energies and inhibition constants of the analogs to aid in the selection of the most favorable analog for chemical synthesis and further examination. Ultimately, the dicoumarin-polyamine (compound **5**) was chosen as the best candidate for ^•^OH detection close to DNA due to its strong DNA-binding, high fluorescence near physiological pH, and linear fluorescence response to radiation doses up to 10 Gy.

**Figure 5:**
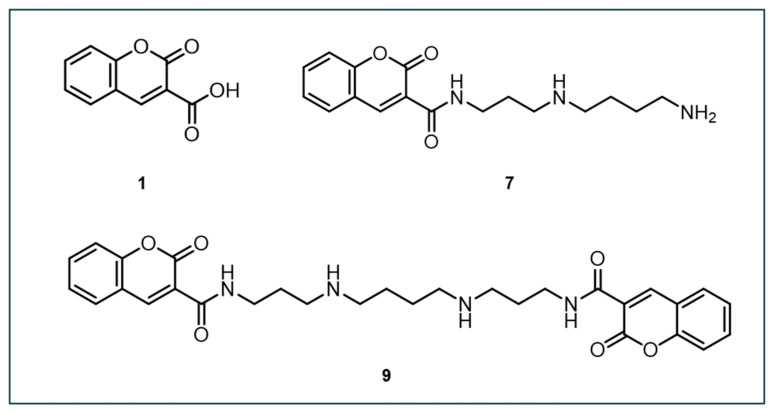
Chemical structures of coumarin-based fluorescent probes: compounds **1**, **7**, and **9**.

In 2008, Singh et al. designed another coumarin-based fluorescent probe, N-(3-(3-aminopropylamino)propyl)-2-oxo-2H-chromene-3-carboxamide (designated as compound **7**), which could potentially bind to DNA and detect ^•^OH (Figure 5) [111]. Compared to other compounds, compound **7** exhibited significantly higher fluorescence yield after hydroxylation, with about three times the QY of CCA (compound **1**) and dicoumarin (compound **9**) at neutral pH. Under acidic conditions, fluorescence induction decreased dramatically. The study demonstrated that the fluorescence signal from compound **7** increased linearly with Na^125^I decay and γ-ray irradiation, saturating at a concentration of ~50 µM. Compound 7 was more sensitive in detecting lower concentrations of ^•^OH, making it potentially useful for detecting ^•^OH near DNA, especially during irradiation.

In 2012, Tang et al. compared CCA with other aromatic chromophores, benzoate, quinolone, and phenoxazine, evaluating their effectiveness as probes in detecting DNA-associated ^•^OH [112]. Benzoate chromophore demonstrated a 17% yield in forming a fluorescent product upon hydroxylation. However, overlapping excitation and emission wavelengths with DNA limit its effectiveness in complex systems. Quinolone and phenoxazine (in its actinomycin D form) avoided this overlap by emitting at longer wavelengths, but had very low hydroxylation yields. Conversely, the CCA system provided a better balance of suitable wavelengths and a moderate yield and remained effective when linked to a DNA-binding peptide. This makes CCA a reliable tool for assessing DNA damage.

### 3.5. Conclusions and Outlook for DNA-Targeting Probes

Hydroxyl radicals cause significant DNA damage that is linked to the pathogenesis of various diseases such as cancer, neurodegenerative disorders, and diabetes. Due to their extremely short lifetimes, accurate detection of DNA-associated ^•^OH in biological systems poses a significant challenge. The urgent need for more sensitive and selective probes is evident, especially those capable of detecting ^•^OH near DNA. Coumarin-based probes, particularly SECCA-labeled histone H1 and nucleosomal histone DNA complexes, show great promise for targeting DNA for the precise detection of DNA-associated ^•^OH. Despite this, several limitations and challenges must be acknowledged and addressed.

#### 3.5.1. Technical Limitations of Coumarin-Based Probes

Coumarin-based probes offer highly sensitive fluorescence detection of ^•^OH but are hindered by several intrinsic limitations. One major limitation is the inner filtering effect, which was first identified by Leandri et al. in 2019. This effect occurs when coumarin absorbs a portion of the excitation light needed for 7-OHC fluorescence, leading to an underestimation of ^•^OH levels at higher coumarin concentrations [113]. The same effect was observed by McCormick et al. (2023) when using coumarin in their novel electrochemical method for monitoring ^•^OH produced during photocatalytic reactions [95]. To mitigate the inner filtering effect, researchers should consider implementing strategies that ensure that fluorescence measurements are more accurate. Currently, the most effective approach is to dilute the sample prior to fluorescence measurements. This reduces the concentration of coumarin and minimizes its absorption of the excitation light needed for 7-OHC fluorescence. While correction factors have been proposed, they can be complex. Dilution is a simple and effective solution which can be used to avoid underestimating ^•^OH levels [114,115].

Another challenge of coumarin-based probes is that they do not directly determine the absolute rate of ^•^OH formation. As shown in the literature, coumarin can react with ^•^OH via multiple pathways, producing at least seven hydroxycoumarin products. Among these, only 7-OHC is strongly fluorescent [94,116]. Since 7-OHC is just one of many products, its fluorescence signal represents only a fraction of the total ^•^OH generated, leading to an underestimation of the ^•^OH formation rate. To accurately calculate the ^•^OH formation rate, it is important to consider the yields of hydroxycoumarin products. Based on the comparison of the yields of 7-OHC and ^•^OH, Zhang and Nosoka (2013) calculated that 6.1% of ^•^OH produced from a photocatalytic reaction can be detected as 7-OHC when using 0.1 mM coumarin [117]. In contrast, Newton and Milligan (2006) reported that 4.7% of ^•^OH generated from water radiolysis can be detected as 7-hydroxycoumarin-3-carboxylic acid (7-OHCCA) using 0.1 mM CCA [118]. Future studies should calibrate coumarin probes under their specific experimental conditions to accurately reflect ^•^OH formation rates. Further research is needed to clarify how factors like concentration, temperature, pH, and ^•^OH generation methods affect detection efficiency.

#### 3.5.2. Probes for Complex Biological Structures

A significant limitation of the SECCA histone H1/polylysine-DNA systems is that they do not accurately replicate the complex geometries of chromatin structures found within living cells. In cells, DNA is packaged into nucleosomes, in which the core DNA is wrapped, in about 1¾ left-handed superhelical turns, around a histone octamer. The nucleosome core is further stabilized by histone H1, which promotes the folding and assembly of higher-order chromatin structures. Adding to this complexity are histone modifications, which influence chromatin dynamics and accessibility. Charkrabarti (1998) made progress in overcoming this limitation by developing a method to label nucleosomal core particles directly with SECCA [107], but further advancements are needed. Efficient labeling of these complex structures is also a challenge. Because the molar ratios of SECCA to histone can significantly impact the QE, accurate and effective labeling is crucial [102]. However, the coupling chemistry of SECCA to larger macromolecules is inefficient, and determining the labeling percentage can be tedious. Future studies should prioritize developing more robust labeling techniques that ensure consistent, high-yield coupling while preserving the biological relevance of the system.

#### 3.5.3. Limitations in Live Cell and In Vivo Applications

To date, DNA-targeting probes have primarily been used in cell-free systems and have not been tested or applied in live cells or in vivo systems. The application of these probes in biological systems presents a new set of challenges, as the complexity of cellular environments and interactions with other biomolecules may interfere with probe performance. Researchers must consider factors such as enzymatic degradation, intracellular localization, and non-specific interactions, all of which can affect probe efficacy and specificity, and the accuracy of signal interpretation. Currently, the feasibility of using these probes in living systems remains uncertain. Further developments will be necessary to enhance the selectivity and stability of probes to withstand the dynamic and complex environments of living systems.

## 4. Fluorescence Detection of Organelle-Associated ^•^OH

Hydroxyl radicals react almost instantaneously at their sites of formation and exist in vivo at picomolar or very low nanomolar concentrations. Consequently, organelles like mitochondria and lysosomes, which are primary sources of ^•^OH, are particularly vulnerable to oxidative damage. Additionally, other cellular regions, such as the nucleus and lipid membranes, are also susceptible to oxidative damage and are implicated in various diseases [119]. To effectively study and understand the impact of ^•^OH in these contexts, it is crucial to design probes that can accurately target these specific cellular sites. These probes must be capable of rapidly reacting with ^•^OH, even in the presence of antioxidants, and produce stable products that can be accurately measured. Such targeted probes will enable a detailed investigation into the role of ^•^OH in cellular processes and the contributions of ^•^OH to disease mechanisms.

### 4.1. Mitochondria-Targeted Probes for ^•^OH Detection

In living biological systems, the mitochondria are a major source of ^•^OH and other ROS produced during aerobic respiration and other metabolic processes. While ROS are important for cellular signaling at low levels, high concentrations can lead to significant oxidative damage. Mitochondrial macromolecules (e.g., DNA, proteins, and lipids) are especially vulnerable due to their close proximity to the site of ROS production. Research has shown that elevated levels of 7,8-dihydro-8-oxo-deoxyguanosine (8-oxo-dG), a marker of oxidative DNA damage, are frequently found in mitochondrial DNA (mtDNA) [120]. Mutations in mtDNA caused by oxidative damage can disrupt the assembly and function of the respiratory chain, leading to the accumulation of ROS. This continuous cycle leads to further oxidative stress, resulting in energy depletion and cell death. Several age-associated human diseases have been linked to mitochondrial dysfunction and oxidative stress, including Alzheimer’s disease, Parkinson’s disease, Huntington’s disease, amyotrophic lateral sclerosis, and cancer [121]. In addition, high production of mitochondrial ROS in hypoxic cells has been linked to angiogenesis-related diseases such as cancers and ischemic disorders [122,123]. To gain a better understanding of the mechanisms of mitochondrial ^•^OH and their role in disease pathogenesis, it is important to develop effective probes that not only target mitochondria specifically but also provide accurate and sensitive measurements of ^•^OH. Over the years, several probes have been developed to accomplish these goals.

#### 4.1.1. Ratiometric Fluorescence Nanosensor CCA@TPP@CDs

**Figure 6:**
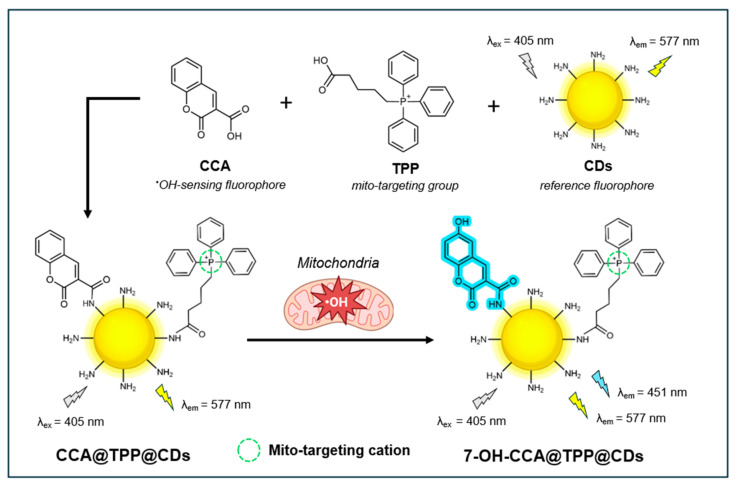
Schematic illustration of the primary components of the CCA@TPP@CDs nanosensor and its application to ^•^OH detection within the mitochondria.

In 2019, Zhou et al. introduced an innovative ratiometric fluorescent nanosensor, termed CCA@TPP@CDs, that leverages carbon nanodots (CDs) to visualize both exogenous and endogenous ^•^OH within the mitochondria of live cells [124]. Through ratiometric detection, this nanosensor improves ^•^OH quantification by providing built-in self-calibration to mitigate the effects of fluctuations in instrumental parameters, inhomogeneous cellular distribution, and photobleaching [125]. As illustrated in Figure 6, the nanosensor combines amino-functionalized CDs with coumarin-3-carboxylic acid (CCA) and (4-carboxybutyl)-triphenylphosphonium bromide (TPP). The CDs provide a stable yellow fluorescence (λ_em_ = 577 nm) to serve as a reliable reference signal, while the CCA emits a blue fluorescence (λ_em_ = 451 nm) as the specific recognition molecule for ^•^OH detection. The distinct separation of the two emission peaks (126 nm) facilitates two-channel ratiometric fluorescence detection of ^•^OH. Through its lipophilic cationic nature, the TPP functions as a targeted ligand for mitochondria. The blue fluorescence at 451 nm gradually increases with increasing ^•^OH concentrations, while the yellow fluorescence from the CDs remains constant. The ratio of the fluorescence intensity levels between 420–500 nm and between 530–610 nm is used to quantify ^•^OH concentrations.

The amine-functionalized CDs were shown to be monodispersed, exhibiting uniform spherical shapes and a narrow diameter distribution (2.1–2.8 nm) with a height of ~2.4 nm. Upon conjugation of CCA and TPP, no obvious effects on size were observed. Dissolved in water, CDs exhibited a fluorescence QY of 12.9%, using rhodamine B as a standard. The CCA@TPP@CDs nanosensor shows high sensitivity for ^•^OH detection, in which the F_blue_/F_yellow_ ratio increases linearly with increasing ^•^OH concentrations (0.1–160 mM) with a limit of detection (LOD) of 70 nM. The probe also demonstrates good selectivity for ^•^OH over other ROS (e.g., O_2_^•−^, H_2_O_2_, HNO, ClO^−^, and ONOO^−^) and biologically relevant metal ions (e.g., K^+^, Na^+^, Ca^2+^, Mg^2+^, and Cu^2+^) at 100 µM concentrations. Additionally, it responds rapidly to ^•^OH, with fluorescence intensities stabilizing within 30 min in the presence of 100 µM ^•^OH. No pH stability or photostability studies were conducted.

In live RAW264.7 cells, CCA@TPP@CDs exhibited good biocompatibility and low cytotoxicity, maintaining 90% viability at a concentration of 120 µg/mL for 48 h. The probe was proven to have excellent mito-targeting abilities, as reflected by the high Pearson’s coefficient (Pr = 0.93) generated from the fluorescence/MitoTracker^TM^ [126] correlation plots. Finally, CCA@TPP@CDs was successfully applied to monitor and image both exogenous and endogenous ^•^OH in the mitochondria, as generated by Fe^2+^/H_2_O_2_ and the apoptotic drug phorbol 12-myristate 13-acetate (PMA), respectively.

#### 4.1.2. Ratiometric Fluorescent Probe NIR-HR

**Figure 7:**
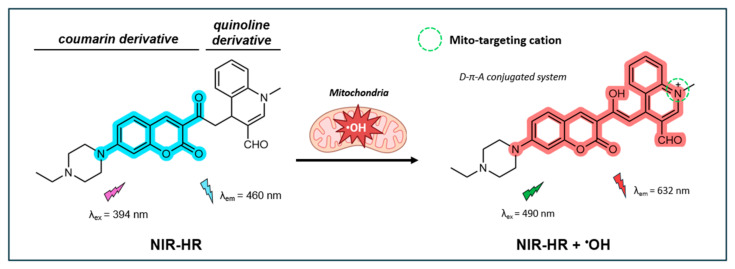
The chemical structure of the dual-emission fluorescent probe NIR-HR and its application to detect ^•^OH localized within the mitochondria.

In a recent study, Ma et al. synthesized a near-infrared (NIR) dual-emission mitochondria-targeting fluorescent probe (NIR-HR) built on a coumarin-quinoline framework [127]. The coumarin component serves as the fluorescent dye, while the quinoline component acts as a proton receptor with increased electron-withdrawing capability upon protonation. This allows for the addition of an aromatic acetyl moiety on the C-4 position, which functions as a recognition group for ^•^OH. While not explicitly stated by the authors, the probe is presumably designed to target mitochondria through hydrophobic interactions facilitated by the inherent alkaloid-like and lipophilic properties of the coumarin-quinoline framework, which is very similar to the probe described by Chen et al. [128]. As illustrated in Figure 7, in its non-oxidative state, the probe exhibits a distinct emission peak at 460 nm when excited at 394 nm. Upon oxidation by ^•^OH, the acetyl group is converted to an enol, resulting in an extended π-conjugated system that follows a donor-π-acceptor (D-π-A) pattern. In this oxidative state, the probe exhibits an emission peak at 632 nm (λ_ex_ = 490 nm). As a result, NIR-HR displays two well-resolved emission peaks with a significant shift of 172 nm. As ^•^OH concentrations increase, the fluorescence signal at 460 nm decreases, while the signal at 632 nm increases. The probe’s NIR fluorescence properties are particularly desirable for bio-system imaging, as they result in less interference from light scattering and tissue autofluorescence.

NIR-HR shows good sensitivity for ^•^OH, demonstrating a linear fluorescence response to ^•^OH concentrations from 1 to 20 µM, with an LOD of 27 nM. NIR-HR shows excellent selectivity for detecting ^•^OH over other common oxidizers (e.g., H_2_O_2_, ONOO^−^, ^1^O_2_, ClO^−^, NO_2_^−^, and tert-butyl hydroperoxide), with negligible interference from common cationic species and amino acids. It also exhibits a rapid response time for ^•^OH sensing, reaching its peak fluorescence intensity within 50 s of the initiation of the Fenton reaction. The calculated fluorescence QY of NIR-HR was not reported. The probe performs well across the pH range from 3 to 8, making it suitable for detecting ^•^OH under physiological conditions. No information was provided on the photostability of the probe.

For practical applications, the NIR-HR probe demonstrates low cytotoxicity in live cells and zebrafish. Immortalized human embryonic kidney cells maintained 84% viability at 30 µM for 24 h. The probe showed a good correlation with the Mito-Tracker Green (Pr = 0.94), indicating good targeting of mitochondria. The probe also showed lysosome-targeting abilities, as reflected by its relatively strong correlation with the Lyso-Tracker Green (Pr = 0.72). This may be due to the tertiary amines present in the probe known to accumulate in lysosomes [129]. In HeLa cells, the probe was successfully used for the real-time tracking of endogenous ^•^OH generated by PMA. The effectiveness of the NIR-HR probe in tracking ^•^OH within organelles was illustrated through experiments with MCF-7 cells to measure apoptosis and autophagy induced by lipopolysaccharides (LPS) and β-lapachone (β-Lap) through ROS generation and accumulation. In the case of β-Lap, NIR-HR showed the capability of tracking ^•^OH in the nuclei of cells in addition to the mitochondria.

#### 4.1.3. Turn-On Fluorescent Probe RThy

**Figure 8:**
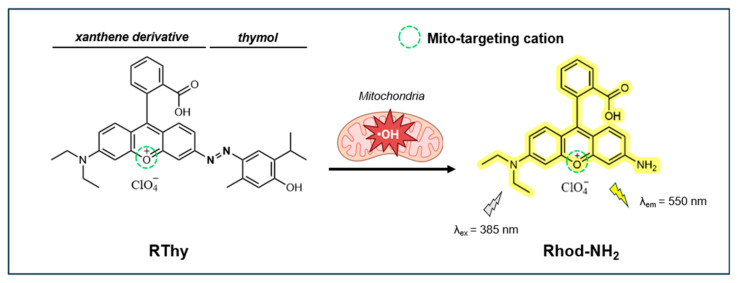
The chemical structure of the Turn-on fluorescent probe RThy and its application to detect ^•^OH localized within the mitochondria.

In 2019, Yuan et al. developed RThy, a novel mitochondria-targeting turn-on xanthene fluorescent probe for detecting ^•^OH in living cells and zebrafish models [130]. As shown in Figure 8, this probe features a thymol molecule linked to a xanthene derivative (Rhod-NH_2_) through the azo bond. The xanthene derivative works as a lipophilic cationic group to allow the probe to specifically target mitochondria. The azo group functions as both a quencher for tunable light absorption and a selective recognition site for ^•^OH. Upon interaction with ^•^OH, the azo bond cleaves to release the fluorophore Rhod-NH_2_, resulting in a substantial increase in fluorescence at 550 nm (λ_ex_ = 385 nm).

RThy exhibits excellent sensitivity to Fe^2+^-catalyzed ^•^OH, showing a strong linear fluorescence response to ^•^OH at concentrations ranging from 0 to 5.0 µM, and an LOD of 8.0 nM. After reacting with ^•^OH, the fluorescence QY of RThy was estimated to be 0.73 in reference to rhodamine 6G in ethanol. In the absence of ^•^OH, RThy is non-fluorescent due to the effective quenching by the azo moiety. An optimal fluorescence response is observed at pH 6.0–8.0, with no significant signal detected in the range of pH 4.0–9.0 in the absence of ^•^OH. The probe also exhibits good photostability under 30 min of radiation from a 100 W mercury lamp at pH 4.5 and 7.4. RThy has excellent selectivity for ^•^OH over other reactive species, including O_2_^•−^, ROO^•^, ClO^−^, NO, ONOO^−^, HNO, ^1^O_2_, H_2_O_2_, Zn^2^⁺, Fe^3+^, and others.

RThy has low cytotoxicity in HeLa cells, with a viability >85% at 20 µM for 24 h. The mitochondria-targeting abilities of RThy are good, as reflected by the high Pearson’s coefficient (Pr = 0.947) between RThy fluorescence and the MitoTracker dye. RThy was effectively applied to detect and image Fe^2+^- and LPS-induced ^•^OH localized within the mitochondria. When applied to zebrafish, RThy produced a bright fluorescence signal in response to Fe^2+^-catalyzed ^•^OH (20 μM). These results highlight the probe’s dual capability of detecting and imaging ^•^OH, making it a valuable tool for studying oxidative stress and related biological processes.

### 4.2. Lysosome-Targeting Fluorescent Probes for ^•^OH Detection

Hydroxyl radicals are among the most prevalent ROS found in lysosomes. Consequently, lysosomes, like mitochondria, are highly susceptible to oxidative damage caused by ^•^OH. Furthermore, the evidence suggests that the lysosome is a major site for basal ROS generation, likely due to the autophagic degradation of ferric proteins, leading to the release of redox-active iron [131]. High levels of ^•^OH (along with other ROS) are among the principal mediators of lysosomal membrane permeabilization (LMP) [132]. LMP is a process of stress-related damage of the lysosomes that results in lysosomal swelling and the subsequent leakage of lysosomal contents into the cytoplasm. Depending on the cell type and extent of cellular damage that occurs, this can trigger apoptosis, pyroptosis, or necrosis [133]. Additionally, through the release of lysosomal cathepsins, LMP can enhance external signals of cell death and hinder cellular recovery through autophagy. LMP is often associated with neurodegenerative disorders such as Alzheimer’s and Parkinson’s [134,135]. Of other interest, the induction of LMP may serve as an effective therapeutic strategy for cancer [136,137]. To properly understand the behavior and functions of ^•^OH in LMP, and to characterize its role in cell death, it is crucial to detect the production of ^•^OH in lysosomes with high specificity and sensitivity.

#### 4.2.1. Two-Photon Turn-On Fluorescent Probe 1-Red

**Figure 9:**
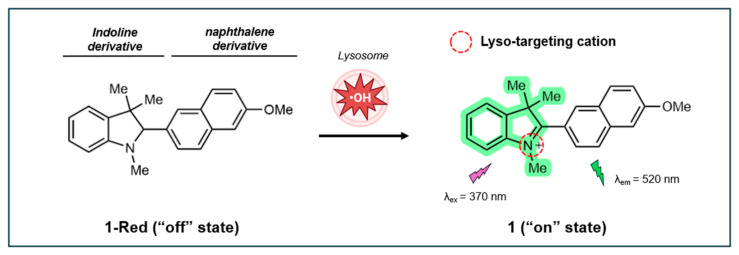
The chemical structure of 1-Red (“off” state) and its application to detect ^•^OH localized within the lysosomes of live cells.

In 2019, Benitez-Martin et al. developed a two-photon (TP) turn-on fluorescent probe based on a naphthalene-indoline compound to detect ^•^OH specifically within the lysosomes of live cells [138]. This probe, referred to as 1-Red in its non-fluorescent “off” state, is equipped with a tertiary amine on the indoline group that targets lysosomes. Once inside the acidic lysosomal environment, 1-Red undergoes oxidation and switches to its “on” state (1), as illustrated in Figure 9. The oxidation of 1-Red restores conjugation between the methoxynaphthalene and the indoline ring, allowing for the fluorescence detection of lysosomal ^•^OH using TP microscopy (λ_ex_ = 740, λ_em_ = 520, cross-section 25 Göppert-Mayer units). The transition results in a remarkable 125-fold increase in fluorescence intensity, which significantly reduces background signals and enhances the signal-to-noise ratio. The oxidation causes the tertiary amine to become protonated, which helps to trap the probe inside the lysosome. This makes the probe highly effective for both in vitro and in vivo imaging studies.

The probe demonstrates good sensitivity to ^•^OH, exhibiting a linear fluorescence response to ^•^OH concentrations ranging from 0.5 to 0.75 µM, with an LOD of 32 nM. Dissolved in water, the probe’s fluorescence QY was estimated to be 0.5, using coumarin 153 (in ethanol) as a reference. Stability studies showed that the probe is resistant to auto-oxidation in acetonitrile and Gly/HCl buffer (pH 2.4) and exhibits high photostability under continuous irradiation for 60 min. Importantly, the probe only undergoes oxidation under acidic conditions and shows high selectivity for ^•^OH over other reactive oxygen and nitrogen species.

In mouse embryonic fibroblasts (MEFs), the probe exhibits reasonably low cytotoxicity at 0.025 µM (cell viability >80%) after 24 h incubation, with significant 1-Red cytotoxicity observed at concentrations above 0.1 µM. The probe effectively detects ^•^OH in MEFs, with strong green fluorescence observed upon activation. The “off” form initially shows minimal fluorescence, which increases over time as it converts to the “on” state. The probe responds well to various ROS-generating agents, including UVA radiation, TBHP, and 2-methoxyestradiol (2-ME), demonstrating its sensitivity to different sources of ROS. Subcellular localization studies reveal that the probe specifically accumulates in lysosomes, showing significant overlap with lysosomal markers (Pr = 0.83) and minimal overlap with mitochondria markers.

#### 4.2.2. Turn-On Fluorescent Probe HCy-Lyso

**Figure 10:**
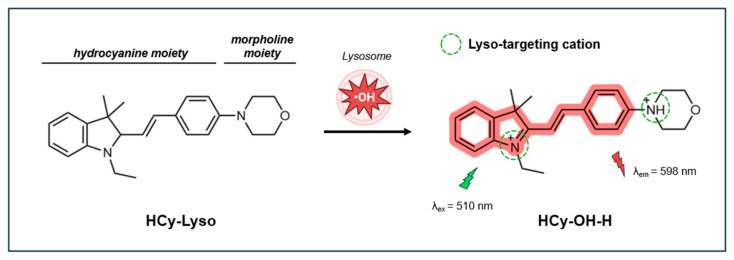
The chemical structure of the fluorescent probe HCy-Lyso and its application to target and detect ^•^OH localized within the lysosomes of live cells.

Recently, Zhong et al. (2024) developed HCy-Lyso, a lysosome-targeting turn-on fluorescent probe based on hydrocyanine [139]. This probe integrates a hydrocyanine moiety for selective recognition of ^•^OH and a morpholine group for lysosome targeting. In the absence of ^•^OH, HCy-Lyso shows minimal fluorescence due to limited π-conjugation and coplanarity. Upon reacting with ^•^OH, the probe undergoes an extension of its π-conjugation system, producing a strong fluorescence signal at 598 nm when excited at 510 nm (Figure 10). Once oxidized, nitrogen in hydrocyanine and the morpholine moieties both become protonated. These protonated amines are membrane-impermeable in the lysosome, resulting in the selective entrapment of the probe in the lysosome.

The probe’s fluorescence response is significantly enhanced under acidic conditions (pH 4.0–7.4), which reflects the acidic environment of lysosomes (pH 4.0–5.5) in living cells. In a pH 4 buffered solution, the fluorescence intensity increases linearly with ^•^OH concentrations up to 10 µM. The fluorescence QY was estimated to be 0.014 in reference to rhodamine B in ethanol. The fluorescence response reaches saturation within 20 min, demonstrating the probe’s rapid reaction to ^•^OH. Importantly, HCy-Lyso is highly selective for ^•^OH over other common ROS such as OCl^−^, ^1^O_2_, NO, H_2_O_2_, and ONOO^−^, as well as TBHP. The photostability of the probe was not reported.

In cells, HCy-Lyso exhibits low cytotoxicity and good biocompatibility, maintaining 90% viability in murine 4T1 breast cancer cells after 12 h of incubation at concentrations up to 10 µM. The probe’s excellent overlap of red fluorescence with the blue fluorescence of LysoTracker (Pr = 0.73) indicates an effective targeting of lysosomes by HCy-Lyso. HCy-Lyso has been successfully employed for imaging and real-time monitoring of endogenous ^•^OH induced by PMA. Additionally, it has been used to track changes in lysosomal ^•^OH levels in two different ferroptosis pathways, namely, those triggered by erastin and (1S, 3R)-RSL3 through inhibition of systems xc− and GPX4, respectively. Treatment with the ferroptosis inhibitor Ferostatin-1 led to a reduction in ^•^OH levels, as evidenced by the decreased fluorescence intensity of HCy-Lyso.

### 4.3. Dual-Organelle-Targeting Fluorescent Probes for ^•^OH Detection

Fluorescent probes that can simultaneously target multiple organelles provide a powerful tool for studying cellular oxidative stress, particularly in the context of disease. Mitochondria and lysosomes, for instance, interact through processes such as mitophagy and mitochondria–lysosome contacts (MLCs), playing an important role in calcium and ion homeostasis [140,141]. The dysfunction of this interaction can result in the development of neurodegenerative diseases such as Parkinson’s disease [142,143]. By targeting two critical organelles at once, these probes allow researchers to observe how two different cellular compartments interact under oxidative stress. This capability could be especially valuable for investigating cellular processes linked to diseases that may induce ^•^OH production in one organelle over another, offering deeper insights into disease mechanisms.

#### 4.3.1. NIR Fluorescent Probe HR-DL

**Figure 11:**
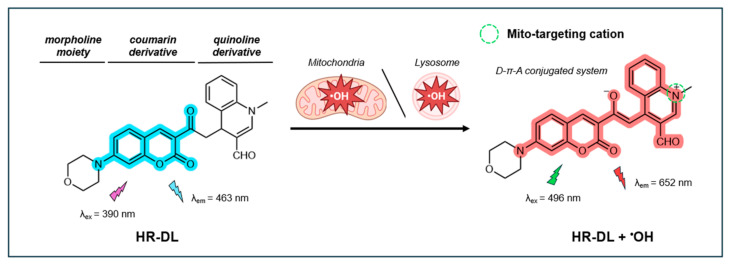
The chemical structure of the fluorescent probe HR-DL and its application to target and detect ^•^OH localized within the mitochondria and lysosomes of live cells.

In 2024, a dual-organelle-targeting NIR fluorescent probe, termed HR-DL, was reported for the simultaneous targeting of mitochondria and lysosomes in live cells [144]. Like the probe NIR-HR designed by Ma et al. [127], HR-DL features a coumarin-quinoline structure with a donor–acceptor (D-π-A) configuration. The quinoline oxidative derivative functions as an electron acceptor and mito-targeting moiety. However, unlike NIR-HR, which contains a 1-ethylpiperazine group, HR-DL incorporates a morpholine moiety as an electron donor and lyso-targeting group. The coumarin fluorophore is positioned between the mito- and lyso-targeting groups, with the acetyl group acting as a selective recognition site for ^•^OH. As illustrated in Figure 11, the probe initially emits a blue fluorescence at 463 nm (λ_ex_ = 390 nm), which undergoes a red-shift in fluorescence upon reacting with ^•^OH, emitting at 652 nm (λ_ex_ = 496 nm).

The fluorescence intensity of HR-DR illustrated good linearity with an ^•^OH concentration up to 20 µM, with an LOD of 39 nM. The fluorescence intensity remains stable within physiological pH (4–8) but is reduced in strongly acidic or basic conditions. The probe demonstrates high selectivity for ^•^OH over other common ROS such as ClO^−^, H_2_O_2_, Fe^2+^, Fe^3+^, and ONOO^−^, as well as various metal ions and bioactive species. The probe’s fluorescence QY and photostability were not reported.

In living cells, the probe exhibits low cytotoxicity, maintaining 80% cell viability at a concentration of 40 µM. HR-DL has been effectively used to monitor and image Fe^2+^-catalyzed ^•^OH in HeLa cells, demonstrating excellent simultaneous targeting of both mitochondria (Pr = 0.91) and lysosomes (Pr = 0.89). The HR-DL has been proven effective in imaging endogenous ^•^OH in zebrafish. Interestingly, this HR-DL probe was also applied to a mouse model to diagnose inflammation through ^•^OH tracking. For this experiment, HR-DL (100 mg/kg, 100 µL) was injected into the peritoneal cavity of the mouse. Liver inflammation was then induced by treatment with LPS (2 mg/kg, 100 µL). The results showed a ~3.1-fold increase in fluorescence intensity in the inflammatory group relative to the control group, underscoring the probe’s potential use as a diagnostic tool for inflammation and as a method for evaluating the therapeutic efficacy of drugs for LPS-induced diseases.

#### 4.3.2. Pyronine-Based Fluorescent Probe PY

**Figure 12:**
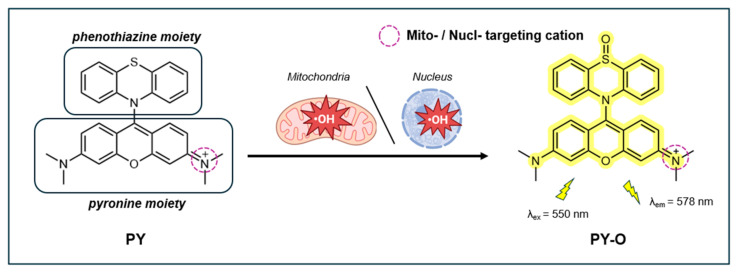
The chemical structure of the fluorescent probe PY and its application to simultaneously detect ^•^OH localized within the mitochondria and nucleoli of live cells.

A fluorescent probe able to detect ^•^OH specifically localized within the mitochondria and nuclei (nucl) using 9-(10H-phenothiazin-10-yl) pyronine (PY) combined with a phenothiazine unit was reported in 2022 by Wang et al. [145]. Pyronine, an intercalating cationic dye, naturally accumulates in the mitochondria and exhibits specificity for RNA [146]. The dual functionality of the pyronine group serves as a fluorophore and the mitochondria and the nuclei targeting group. Phenothiazine, a heterocyclic compound, functions a group detecting ^•^OH. The probe exists in two states: the non-fluorescent form (PY) and the fluorescent form (PY-O, 9-(5-oxido 10H-phenothiazin-10-yl) pyronine), where the latter indicates the presence of oxidative stress (Figure 12).

The probe, exhibiting minimal fluorescence due to photo-induced electron transfer (PET), showed a significant fluorescence emission at 578 nm (λ_ex_ = 550 nm) upon reaction with ^•^OH. The fluorescence intensity had an excellent direct relationship with ^•^OH concentrations up to 20.0 μM, with an LOD of 13.6 nM. Its high sensitivity is accompanied by a rapid response time of <1 s. PY displayed high selectivity for ^•^OH over other ROS, RNS, RSS, and Fe^2+^/Fe^3+^ ions. Moreover, the fluorescence intensity of PY was not significantly affected by pH changes in the range of 4.0–9.0 or by external stimuli such as LPS, Tempo, erastin, Fer-1, β-Lab, rapamycin, and BLM. The fluorescence QY and photostability of the probe were not reported.

PY exhibits low cytotoxicity in A549 and HeLa cells, with >90% viability at 50.0 µM. It has good cell permeability and mitochondria-targeting ability in both cell lines, with high Pearson coefficients (Pr > 0.90) reported between the probe’s red fluorescence signal and the Mito Green Tracker. The authors did not provide a Pearson’s coefficient to reflect the probe’s effectiveness in targeting the nucleus. PY was successfully applied in A549 and HeLA cells to monitor the changes in endogenous ^•^OH levels in response to LPS and the ^•^OH scavenger Tempo. In erastin/bleomycin-treated A549 cells undergoing ferroptosis, PY effectively monitored intracellular ^•^OH levels in both the mitochondria and nucleus. PY was also applied to detect ^•^OH generated during other forms of cell death, such as autophagy (induced by rapamycin) and apoptosis (induced by β-Lap). Interestingly, the probe revealed that rapamycin and β-Lap did not induce ferroptosis, but only induced ^•^OH production in the mitochondria.

### 4.4. Lipid Membrane-Targeting Fluorescent Probes for ^•^OH Detection

Probes that specifically target lipid membranes are valuable for studying oxidative stress, as they allow for real-time monitoring of lipid peroxidation and its impact on membrane integrity. Lipid peroxidation, a key process in cellular oxidative stress, results in a breakdown of membrane lipids, which compromises cell integrity and function, ultimately leading to programmed cell-death. ROS-induced lipid peroxidation has been shown to promote apoptosis, autophagy, and ferroptosis [147], and is implicated in the pathogenesis of diseases such as atherosclerosis, carcinogenesis, and diabetes mellitus [148,149,150]. Probes that target lipid membranes can directly detect the initiation and progression of lipid peroxidation, providing a deeper understanding of their roles in disease pathology, and facilitating identification of potential therapeutic targets which might be used to mitigate oxidative damage.

#### CCA-Based Fluorescent Probe DPPEC

**Figure 13:**
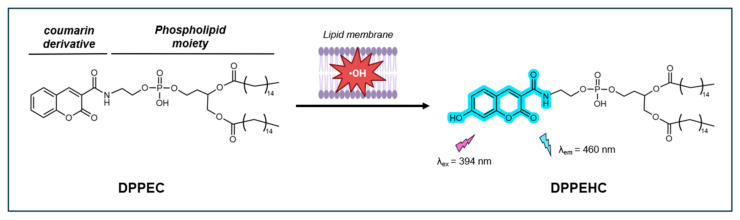
The chemical structure of the fluorescent probe DPPEC and its application to detect ^•^OH localized within lipid membranes of live cells.

In 2008, Soh et al. introduced DPPEC, a new fluorescent probe for detecting ^•^OH in lipid membranes [100]. This probe consists of two key components: CCA and a phospholipid 1,2-dipalmitoylglycerophosphorylethanolamine (DPPE). CCA specifically recognizes and reacts with ^•^OH, while DPPE anchors itself within the lipid membranes. Upon reacting with ^•^OH, CCA is converted to the highly fluorescent 7-OHCCA (λ_ex_ = 400 nm, λ_em_ = 444 nm) (Figure 13).

The fluorescence of DPPEC was shown to increase proportionally with increasing concentrations of CuSO_4_ ranging from 0 to 10 µM, suggesting that the probe efficiently responds to increasing ^•^OH concentrations. Furthermore, the probe showed good selectivity for ^•^OH against other ROS such as H_2_O_2_, O_2_^•−^, NO, ONOO^−^, and OCl^−^, and did not respond to the separate addition of CuSO_4_ and ascorbic acid. Notably, the fluorescence QY, LOD, pH stability, and photostability of this probe were not reported.

DPPEC demonstrated effective localization within lipid membranes in live RAW 264 cells, allowing for successful detection of ^•^OH generated by the CuSO_4_/H_2_O_2_/ascorbic acid system. The detection of ^•^OH was confirmed by the loss of fluorescence when experiments were conducted in the presence of DMSO. However, the cytotoxicity of DPPEC was not reported. Through the effective monitoring of ^•^OH in lipid membranes, this probe has the potential to aid in the investigation of lipid peroxidation, one of the most significant biological processes involving ^•^OH.

### 4.5. Conclusions and Outlook for Organelle-Targeting Probes

Intracellular organelles such as mitochondria, lysosomes, and nuclei, as well as lipid membranes, are particularly vulnerable to the oxidative damage caused by ^•^OH. Such damage contributes to the oxidative stress, a key factor in various age-related diseases. Numerous fluorescent probes have been developed to detect and monitor organelle-associated ^•^OH, and are summarized in this work. Researchers have incorporated targeting moieties such as TPP, morpholine, and DPPE to specifically target mitochondria, lysosomes, and lipid membranes, respectively (Table 2). Although these probes hold promise for organelle-targeted ^•^OH detection, several limitations and inconsistencies must be addressed.

#### 4.5.1. Establishing Robust Characterization of Probes

A thorough review of the literature highlights the need for a standardized characterization protocol for organelle-targeting fluorescent probes designed to detect ^•^OH. Key parameters like fluorescence quantum yield (QY), limit of detection (LOD), and photostability are all essential for evaluating probe performance but are not uniformly reported across studies. In particular, steps should be taken to ensure that QY determination methods are accurate and consistent across studies. Nawara and Waluk (2017) suggested an improved method of QY determination using the simultaneous absorption and fluorescence emission (SAFE) measurement obtained using a single commercial spectrofluorometer [151]. For biological-imaging applications, photostability is a particularly important characteristic, especially for long-wavelength probes [152]. Beyond these parameters, consistent evaluation of selectivity, targeting specificity, and live-cell compatibility is also critical to optimizing probe performance in biological systems. Establishing a standardized protocol that incorporates these factors can facilitate reliable comparisons of probes and guide the refinement of this technique for enhanced organelle-specific ^•^OH detection.

#### 4.5.2. Achieving Target Specificity for Organelles

Designing probes with high specificity for a single organelle is a significant challenge. Building a successful probe requires clear understanding of the molecular interactions and sub-cellular targeting mechanisms of each organelle.

As previously mentioned, delocalized lipophilic cations (DLCs) such as TPP are frequently used as targeting moieties for the mitochondria. These cations are naturally attracted to the highly negative mitochondrial membrane potential, allowing passive diffusion and accumulation in mitochondria [153]. However, one key limitation of DLCs is that they are entirely reliant upon the maintenance of the mitochondrial membrane potential, which may become problematic in conditions like ischemia, where the mitochondrial membrane potential is unstable [154]. It should also be noted that, at high concentrations, DLCs can cause the depolarization of the negative mitochondrial membrane potential or may interfere with various complexes in the ETC [155]. To avoid cytotoxicity and ensure long-term retention within mitochondria, it may be necessary to incorporate additional moieties that allow covalent attachment. Researchers may also consider utilizing alternative targeting mechanisms of the mitochondria, such as the mitochondrial protein import mechanism using mitochondria-penetrating peptides (MPPs) [156].

Lipophilic amines like morpholine and dimethylamine are the targeting moieties most commonly used for lysosomes, as highlighted above. This strategy exploits the steep pH gradient between the lysosomal lumen and the cytosol, promoting the accumulation of small amine-containing molecules via pH partitioning [157]. Once the molecule enters the lysosome, the amine group is protonated, ensuring the probe’s retention and preventing its efflux. However, these amine-based compounds have specific limitations. Notably, lysosomes are not the only acidic subcellular component. Endosomes also exhibit acidity and can sequester basic amines, potentially reducing targeting lysosomal specificity. It should also be noted that the accumulation of lipophilic alkaline moieties can increase lysosomal pH, which in turn may lead to high pH-induced apoptosis [158,159]. Furthermore, hydrolytic enzymes within the lysosome can degrade fluorescent probes, resulting in misleading losses of fluorescent signals. To ensure signal accuracy, researchers should consider strategies to stabilize their probes within the lysosomal environment.

Amongst the existing ^•^OH-detecting probes, there is a noticeable lack of probes targeting organelles such as the nucleus, ER, and Golgi apparatus. This is mainly due to the limited targeting strategies available for these organelles. While mitochondrial and lysosomal targeting are better developed, further exploration of new targeting strategies directed towards other organelles is needed.

#### 4.5.3. Enhancing Bioimaging Capabilities

To expand the biomedical potential of the fluorescent probes discussed in this review, one promising direction involves integrating nano-platforms such as quantum dots (QDs), metal nanoclusters, polymeric nanoparticles (NPs), and other NPs into the probe. Compared to traditional molecular probes, NPs tend to have higher photostability and reduced risk of non-specific binding to biomacromolecules [160]. Also, by optimizing their charge and surface chemistry, these particles can be easily internalized [161]. Coupling this capability with organelle-targeting moieties may greatly enhance the effectiveness of selective organelle targeting. As reviewed by Hou et al., numerous NP-based fluorescent probes have been developed for the detection of ^•^OH [40]. However, none are equipped to specifically target DNA or organelles, limiting the ability of these probes to provide detailed insights into the variations of ^•^OH in pathological processes.

The fluorescent probes based on coumarin, xanthene, and pyronine tend to exhibit short excitation (≤550 nm) and emission (≤577 nm) wavelengths. This may restrict tissue penetration depth, reduce signal-to-noise ratio, and increase the risk of phototoxicity, thereby limiting their applications in bioimaging [162]. To facilitate in vivo imaging of ^•^OH dynamics, further exploration into the application of NIR fluorescent probes is recommended.

#### 4.5.4. Improving Limits of Detection

Due to their short lifetimes, hydroxyl radicals are present in vivo at picomolar to very low nanomolar concentrations. While the lowest reported LOD of the probes reviewed, 8.0 nM [130], represents significant progress in detection capabilities, there is still substantial room for improvement to achieve sensitivity at physiologically relevant levels. To improve the LOD, researchers can explore strategies to further minimize background noise by reducing interference from other ROS. Additionally, applying filter-optimization methods to improve the signal-to-noise ratio (SNR) can reduce optical background noise [163]. The integration of nanoparticles may also significantly improve detection limits [164], allowing for more sensitive detection of hydroxyl radicals in vivo.

## 5. Development of ^•^OH-Detecting Probes for Real-Life Biomedical Applications

The fluorescent probes discussed in this review are crucial for advancing our understanding of the roles of ^•^OH in both physiological and pathological processes. By enabling specific targeting of DNA and various organelles, these probes could provide a more detailed, localized view of ^•^OH variations within living systems. This capability makes them highly promising as diagnostic tools for oxidative stress-related diseases and could facilitate early detection and monitoring of conditions influenced by ^•^OH.

As reported by Chakrabarti et al., DNA-targeted ^•^OH probes offer valuable insights into the site-specific formation of ^•^OH and the oxidative damage induced by metal-bound chemotherapeutic drugs such as bleomycin and Adriamycin [103,104]. In radiotherapy, these probes can be used to monitor ^•^OH generation and DNA damage induced by radiation and can enable real-time assessment of the efficacy of antioxidants or other treatments aimed at mitigating oxidative DNA damage in normal tissues. These probes can also be valuable tools for elucidating how ^•^OH-induced DNA damage contributes to the progression of various diseases such as cancer, neurodegenerative disorders, and inflammatory conditions.

Organelle-targeted ^•^OH probes are valuable tools for developing organelle-specific oxidative stress profiles to aid in our understanding of disease mechanisms. For instance, they can aid in investigating the role of ^•^OH in mitochondria-lysosome intercommunication under oxidative stress [165], a process linked to the progression of degenerative diseases [166]. These probes also hold the potential to facilitate the discovery and development of antioxidants designed to target and protect specific organelles from ^•^OH-induced damage [167,168]. Organelle-targeted ^•^OH probes may also serve as a tool to evaluate the potential toxicity of certain drug delivery systems, such as silver nanoparticles, and assess the efficacy of potential protective compounds [169].

A major obstacle in advancing the use of newly developed fluorescent probes is the need to ensure that they are validated across diverse biological systems. Despite their potential advantages, probes in development are often overlooked in favor of already established commercial alternatives. To overcome this challenge, researchers must prioritize thorough validation studies and encourage collaboration to ensure the successful translation of ^•^OH-detecting probes from development to practical, real-world applications.

## Figures and Tables

**Table 1:** Key criteria of fluorescent probes for ^•^OH detection.

Key Criteria of ^•^OH Probe	Purpose
High selectivity/specificity	To ensure accurate and specific detection of ^•^OH without interference from other ROS.
High sensitivity	To detect low concentrations of ^•^OH, improving the ability to observe subtle changes in cellular environments.
High photostability	To resist photobleaching during illumination to maintain consistent, reliable detection of ^•^OH over extended time periods.
Stability across biologically relevant pH range	To maintain consistent performance across different pH levels, ensuring reliability in various biological settings.
Resistance to interference	To avoid inaccurate measurements caused by radiation, transition metals, proteins, or ions, preserving the probe’s integrity.
Effective targeting and retention	To enable detection at the precise location within the cell, minimizing background interference from the solution.
Non-altering binding	To ensure the probe binds to target macromolecules, such as DNA, without altering their structure or compromising functionality.
Cell permeability	To facilitate quick and easy entry into cells for effective targeting at cellular sites.
Low cytotoxicity	To minimize harmful effects on cells, allowing for more reliable in vitro and in vivo experiments.

**Table 2:** Summary of organelle-targeting probes, their targeting groups, and their key advantages and limitations.

Ref.	Probe Name	Organelle	Targeting Group(s)	Key Advantage(s)	Key Limitation(s)
[124]	CCA@TPP@CDs	Mitochondria	TPP *	Ratiometric detection High sensitivity High selectivity Rapid response rate High QY * Low cytotoxicity Good mito-targeting ability	No report of photostability Inner filtering effect
[127]	NIR-HR	Mitochondria	Coumarin-quinoline	Ratiometric detection NIR * fluorescence High sensitivity High selectivity Rapid response rate	Off-target accumulation in lysosomes/nuclei No report of QY * or photostability Inner filtering effect
[130]	RThy	Mitochondria	Xanthene derivative	High sensitivity High selectivity High QY * High photostability Stable at physiological pH Good lyso-targeting ability	None specified
[138]	1-Red	Lysosome	Indoline derivative	TP * fluorescence High sensitivity High selectivity High QY * High photostability Sensitive to lysosomal pH Good lyso-targeting ability	Exhibits significant cytotoxicity above 0.1 µM
[139]	HCy-Lyso	Lysosome	Morpholine	High sensitivity High selectivity Sensitive to lysosomal pH Rapid response rate Low cytotoxicity Good lyso-targeting ability	No report of photostability
[144]	HR-DL	Mitochondria, lysosome	Quinoline, morpholine	NIR fluorescence properties High sensitivity High selectivity Stable at physiological pH Low cytotoxicity Good mito- and lyso-targeting abilities	No report of QY * or photostability Inner filtering effect
[145]	PY	Mitochondria, nucleoli	Pyronine	High sensitivity High selectivity Rapid response rate Stable across wide pH range Low cytotoxicity High mito-targeting ability	No report of QY * or photostability
[100]	DPPEC	Lipid membrane	DPPE *	High selectivity Good membrane-targeting ability	No report of QY *, LOD *, photostability, or cytotoxicity Inner filtering effect

* Abbreviations: TPP, triphenylphosphonium; QY, quantum yield; NIR, near-infrared; TP, two-photon; DPPE, 1,2-dipalmitoylglycerophosphorylethanolamine; LOD, limit of detection.

## References

[1] Phaniendra A., Jestadi D.B., Periyasamy L. (2015). Free Radicals: Properties, Sources, Targets, and Their Implication in Various Diseases. Indian J. Clin. Biochem..

[2] Cadenas E., Davies K.J.A. (2000). Mitochondrial free radical generation, oxidative stress, and aging. Free Radic. Biol. Med..

[3] Lambert A.J., Brand M.D., Stuart J.A. (2009). Reactive oxygen species production by mitochondria. Mitochondrial DNA.

[4] Turrens J.F. (2003). Mitochondrial formation of reactive oxygen species. J. Physiol..

[5] Zeeshan H.M.A., Lee G.H., Kim H.R., Chae H.J. (2016). Endoplasmic reticulum stress and associated ROS. Int. J. Mol. Sci..

[6] Chandel N.S. (2021). Nadph—The forgotten reducing equivalent. Cold Spring Harb. Perspect. Biol..

[7] Hong Y., Boiti A., Vallone D., Foulkes N.S. (2024). Reactive Oxygen Species Signaling and Oxidative Stress: Transcriptional Regulation and Evolution. Antioxidants.

[8] Di Meo S., Reed T.T., Venditti P., Victor V.M. (2016). Role of ROS and RNS Sources in Physiological and Pathological Conditions. Oxid. Med. Cell. Longev..

[9] Faraci F.M. (2006). Reactive oxygen species: Influence on cerebral vascular tone. J. Appl. Physiol..

[10] Lee J., Song C.-H., Filosa S. (2021). Effect of Reactive Oxygen Species on the Endoplasmic Reticulum and Mitochondria during Intracellular Pathogen Infection of Mammalian Cells. Antioxidants.

[11] Powers S.K., Jackson M.J. (2008). Exercise-Induced Oxidative Stress: Cellular Mechanisms and Impact on Muscle Force Production. Physiol. Rev..

[12] Dupré-Crochet S., Erard M., Nüβe O. (2013). ROS production in phagocytes: Why, when, and where?. J. Leukoc. Biol..

[13] Lushchak V.I. (2014). Free radicals, reactive oxygen species, oxidative stress and its classification. Chem. Biol. Interact.

[14] Jackson A.L., Loeb L.A. (2001). The contribution of endogenous sources of DNA damage to the multiple mutations in cancer. Mutat. Res..

[15] Wadhwa N., Mathew B.B., Jatawa S.K., Tiwari A. (2012). Lipid peroxidation: Mechanism, models and significance. Int. J. Curr. Sci..

[16] Stadtman E.R., Levine R.L. (2000). Protein oxidation. Ann. N. Y. Acad. Sci..

[17] Hajam Y.A., Rani R., Ganie S.Y., Sheikh T.A., Javaid D., Qadri S.S., Pramodh S., Alsulimani A., Alkhanani M.F., Harakeh S. (2022). Oxidative Stress in Human Pathology and Aging: Molecular Mechanisms and Perspectives. Cells.

[18] Rahman T., Hosen I., Islam M.M.T., Shekhar H.U. (2012). Oxidative stress and human health. Adv. Biosci. Biotechnol..

[19] Kim M., Han C.H., Lee M.Y. (2014). NADPH oxidase and the cardiovascular toxicity associated with smoking. Toxicol. Res..

[20] Leach J.K., Tuyle G.V., Lin P.-S., Schmidt-Ullrich R., Mikkelsen R.B. (2001). Ionizing Radiation-induced, Mitochondria-dependent Generation of Reactive Oxygen/Nitrogen 1. Cancer Res..

[21] Sun Q., Long Z., Wu H., Liu Y., Wang L., Zhang X., Wang X., Hai C. (2015). Effect of alcohol on diethylnitrosamine-induced hepatic toxicity: Critical role of ROS, lipid accumulation, and mitochondrial dysfunction. Exp. Toxicol. Pathol..

[22] Bianco C.L., Toscano J.P., Fukuto J.M., Ignarro L.J., Freeman B.A. (2017). An Integrated View of the Chemical Biology of NO, CO, H_2_S, and O_2_. Nitric Oxide.

[23] Koppenol W.H., Stanbury D.M., Bounds P.L. (2010). Electrode potentials of partially reduced oxygen species, from dioxygen to water. Free Radic. Biol. Med..

[24] Buxton G.V. (1988). Critical Review of Rate Constants for Reactions of Hydrated Electrons, Hydrogen Atoms and Hydroxyl Radicals. J. Phys. Chem. Ref. Data.

[25] Root R., Okada S. (1975). Estimation of life times and diffusion distances of radicals involved in X-ray-induced DNA strand breaks or killing of mammalian cells. Radiat. Res..

[26] Gligorovski S., Strekowski R., Barbati S., Vione D. (2015). Environmental Implications of Hydroxyl Radicals (•OH). Chem. Rev..

[27] Rosen H., Klebanoff S.J. (1979). Hydroxyl Radical Generation by Polymorphonuclear Leukocytes Measured by Electron Spin Resonance Spectroscopy. J. Clin. Investig..

[28] Vásquez-Vivar J., Kalyanaraman B., Kennedy M.C. (2000). Mitochondrial aconitase is a source of hydroxyl radical. An electron spin resonance investigation. J. Biol. Chem..

[29] Peralta E., Roa G., Hernandez-Servin J.A., Romero R., Balderas P., Natividad R. (2014). Hydroxyl Radicals quantification by UV spectrophotometry. Electrochim. Acta.

[30] Zhao H., Gao J., Zhou W., Wang Z., Wu S. (2015). Quantitative detection of hydroxyl radicals in Fenton system by UV-vis spectrophotometry. Anal. Methods.

[31] Cui H., Ma J., Liu Y., Wang C., Song Q. (2024). Dimethyl Sulfoxide: An Ideal Electrochemical Probe for Hydroxyl Radical Detection. ACS Sens..

[32] Li Y., Li Y., Zhang Y., Song Y., Jiang Y. (2019). A rapid and sensitive electrochemical sensor for hydroxyl free radicals based on self-assembled monolayers of carboxyl functionalized graphene. J. Solid. State Electrochem..

[33] Jen J.-F., Leu M.-F., Yang T.C. (1998). Determination of hydroxyl radicals in an advanced oxidation process with salicylic acid trapping and liquid chromatography. J. Chromatogr. A.

[34] Cao Y., Sui D., Zhou W., Lu C. (2016). Highly selective chemiluminescence detection of hydroxyl radical via increased π-electron densities of rhodamine B on montmorillonite matrix. Sens. Actuators B Chem..

[35] Miller C.J., Rose A.L., Waite T.D. (2011). Phthalhydrazide chemiluminescence method for determination of hydroxyl radical production: Modifications and adaptations for use in natural systems. Anal. Chem..

[36] Ganea G.M., Kolic P.E., El-Zahab B., Warner I.M. (2011). Ratiometric coumarin−neutral red (CONER) nanoprobe for detection of hydroxyl radicals. Anal. Chem..

[37] Zhuang M., Ding C., Zhu A., Tian Y. (2014). Ratiometric Fluorescence Probe for Monitoring Hydroxyl Radical in Live Cells Based on Gold Nanoclusters. Anal. Chem..

[38] Wu J., Zhao Y., Li K., Muhammad S., Ju M., Liu L., Huang Y., Wang B., Ding W., Shen B. (2022). Fluorogenic toolbox for facile detecting of hydroxyl radicals: From designing principles to diagnostics applications. Trends Analyt. Chem..

[39] Alanazi M., Yong J., Wu M., Zhang Z., Tian D., Zhang R. (2024). Recent Advances in Detection of Hydroxyl Radical by Responsive Fluorescence Nanoprobes. Chem. Asian J..

[40] Hou J.T., Zhang M., Liu Y., Ma X., Duan R., Cao X., Yuan F., Liao Y.X., Wang S., Xiu Ren W. (2020). Fluorescent detectors for hydroxyl radical and their applications in bioimaging: A review. Coord. Chem. Rev..

[41] Żamojć K., Zdrowowicz M., Jacewicz D., Wyrzykowski D., Chmurzyński L. (2016). Fluorescent and Luminescent Probes for Monitoring Hydroxyl Radical under Biological Conditions. Crit. Rev. Anal. Chem..

[42] Lloyd R.V., Hanna P.M., Mason R.P. (1997). The origin of the hydroxyl radical oxygen in the Fenton reaction. Free Radic. Biol. Med..

[43] Gaware V., Kotade K., Dhamak K., Somawanshi S. (2010). Ceruloplasmin its role and significance: A review. Pathology.

[44] Linder C.M., Wooten L., Cerveza P., Cotton S., Shulze R., Lomeli N. (1998). Copper transport. Am. J. Clin. Nutr..

[45] Munro H.N. (1990). Iron regulation of ferritin gene expression. J. Cell Biochem..

[46] Plays M., Müller S., Rodriguez R. (2021). Chemistry and biology of ferritin. Metallomics.

[47] Agrawal R., Sharma P.K., Rao G.S. (2001). Release of iron from ferritin by metabolites of benzene and superoxide radical generating agents. Toxicology.

[48] Bolann B.J., Ulvik R.J. (1987). Release of iron from ferritin by xanthine oxidase Role of the superoxide radical. Biochem. J..

[49] Thomas C.E., Morehouse L.A., Austs S.D. (1985). Ferritin and Superoxide-dependent Lipid Peroxidation. J. Biol. Chem..

[50] Fang X., Ardehali H., Min J., Wang F. (2023). The molecular and metabolic landscape of iron and ferroptosis in cardiovascular disease. Nat. Rev. Cardiol..

[51] Su L.J., Zhang J.H., Gomez H., Murugan R., Hong X., Xu D., Jiang F., Peng Z.Y. (2019). Reactive Oxygen Species-Induced Lipid Peroxidation in Apoptosis, Autophagy, and Ferroptosis. Oxid. Med. Cell. Longev..

[52] Ma J., Denisov S.A., Adhikary A., Mostafavi M. (2019). Ultrafast processes occurring in radiolysis of highly concentrated solutions of nucleosides/tides. J. Mol. Sci..

[53] Brand M.D. (2010). The sites and topology of mitochondrial superoxide production. Exp. Gerontol..

[54] Aon M.A., Stanley B.A., Sivakumaran V., Kembro J.M., O’Rourke B., Paolocci N., Cortassa S. (2012). Glutathione/thioredoxin systems modulate mitochondrial H_2_O_2_ emission: An experimental-computational study. J. Gen. Physiol..

[55] He L., He T., Farrar S., Ji L., Liu T., Ma X. (2017). Antioxidants Maintain Cellular Redox Homeostasis by Elimination of Reactive Oxygen Species. Cell Physiol. Biochem..

[56] Rueda C.B., Llorente-Folch I., Amigo I., Contreras L., González-Sánchez P., Martínez-Valero P., Juaristi I., Pardo B., Del Arco A., Satrústegui J. (2014). Ca^2+^ regulation of mitochondrial function in neurons. Biochim. Biophys. Acta Bioenerg..

[57] Wang X., Zheng W. (2019). Ca^2+^ homeostasis dysregulation in Alzheimer’s disease: A focus on plasma membrane and cell organelles. FASEB J..

[58] Baumgartner H.K., Gerasimenko J.V., Thorne C., Ferdek P., Pozzan T., Tepikin A.V., Petersen O.H., Sutton R., Watson A.J.M., Gerasimenko O.V. (2009). Calcium elevation in mitochondria is the main Ca^2+^ requirement for mitochondrial permeability transition pore (mPTP) opening. J. Biol. Chem..

[59] Calvo-Rodriguez M., Hou S.S., Snyder A.C., Kharitonova E.K., Russ A.N., Das S., Fan Z., Muzikansky A., Garcia-Alloza M., Serrano-Pozo A. (2020). Increased mitochondrial calcium levels associated with neuronal death in a mouse model of Alzheimer’s disease. Nat. Commun..

[60] Kawamata H., Manfredi G. (2010). Mitochondrial dysfunction and intracellular calcium dysregulation in ALS. Mech. Ageing Dev..

[61] Santulli G., Xie W., Reiken S.R., Marks A.R. (2015). Mitochondrial calcium overload is a key determinant in heart failure. Proc. Natl. Acad. Sci. USA.

[62] Diaz B., Shani G., Pass I., Anderson D., Quintavalle M., Courtneidge S.A. (2009). Tks5-dependent, nox-mediated generation of reactive oxygen species is necessary for invadopodia formation. Sci. Signal.

[63] Graham K.A., Kulawiec M., Owens K.M., Li X., Desouki M.M., Chandra D., Singh K.K. (2010). NADPH oxidase 4 is an oncoprotein localized to mitochondria. Cancer Biol. Ther..

[64] Hilenski L.L., Clempus R.E., Quinn M.T., Lambeth J.D., Griendling K.K. (2004). Distinct Subcellular Localizations of Nox1 and Nox4 in Vascular Smooth Muscle Cells. Arterioscler. Thromb. Vasc. Biol..

[65] Kuroda J., Nakagawa K., Yamasaki T., Nakamura K.I., Takeya R., Kuribayashi F., Imajoh-Ohmi S., Igarashi K., Shibata Y., Sueishi K. (2005). The superoxide-producing NAD(P)H oxidase Nox4 in the nucleus of human vascular endothelial cells. Genes Cells.

[66] Petry A., Djordjevic T., Weitnauer M., Kietzmann T., Hess J., Gӧrlach A. (2006). NOX2 and NOX4 Mediate Proliferative Response in Endothelial Cells. Antioxid. Redox Signal..

[67] Yang B., Rizzo V. (2007). TNF-potentiates protein-tyrosine nitration through activation of NADPH oxidase and eNOS localized in membrane rafts and caveolae of bovine aortic endothelial cells. Am. J. Physiol. Heart Circ. Physiol..

[68] Vermot A., Petit-Hartlein I., Smith S.M.E., Fieschi F. (2021). NADPH Oxidases (NOX): An Overview from Discovery, Molecular Mechanisms to Physiology and Pathology. Antioxidants.

[69] Kleinschnitz C., Grund H., Wingler K., Armitage M.E., Jones E., Mittal M., Barit D., Schwarz T., Geis C., Kraft P. (2010). Post-stroke inhibition of induced NADPH Oxidase type 4 prevents oxidative stress and neurodegeneration. PLoS Biol..

[70] Rastogi R., Geng X., Li F., Ding Y. (2017). NOX activation by subunit interaction and underlying mechanisms in disease. Front. Cell. Neurosci..

[71] Sharma N., Kapoor M., Nehru B. (2016). Apocyanin, NADPH oxidase inhibitor prevents lipopolysaccharide induced α-synuclein aggregation and ameliorates motor function deficits in rats: Possible role of biochemical and inflammatory alterations. Behav. Brain Res..

[72] Fery-Forgues S., Lavabre D. (1999). Are Fluorescence Quantum Yields So Tricky to Measure? A Demonstration Using Familiar Stationery Products. J. Chem. Educ..

[73] Cooke M.S., Evans M.D., Dizdaroglu M., Lunec J. (2003). Oxidative DNA damage: Mechanisms, mutation, and disease. FASEB J..

[74] Whiteman M., Shan Hong H., Jenner A., Halliwell B. (2002). Loss of oxidized and chlorinated bases in DNA treated with reactive oxygen species: Implications for assessment of oxidative damage in vivo. Biochem. Biophys. Res. Commun..

[75] Lloyd D.R., Phillips D.H., Carmichael P.L. (1997). Generation of Putative Intrastrand Cross-Links and Strand Breaks in DNA by Transition Metal Ion-Mediated Oxygen Radical Attack. Chem. Res. Toxicol..

[76] Clayton D.A., Doda J.N., Friedberg E.C. (1974). The Absence of a Pyrimidine Dimer Repair Mechanism in Mammalian Mitochondria. Proc. Natl. Acad. Sci. USA.

[77] Barker S., Weinfeld M., Murray D. (2005). DNA-protein crosslinks: Their induction, repair, and biological consequences. Mutat. Res. Rev. Mutat. Res..

[78] Ding H., Greenberg M.M. (2007). λ-radiolysis and hydroxyl radical produce interstrand cross-links in DNA involving thymidine. Chem. Res. Toxicol..

[79] Cadet J., Wagner J.R. (2014). Oxidatively generated base damage to cellular DNA by hydroxyl radical and one-electron oxidants: Similarities and differences. Arch. Biochem. Biophys..

[80] Cadet J., Wagner J.R. (2013). DNA base damage by reactive oxygen species, oxidizing agents, and UV radiation. Cold Spring Harb. Perspect. Biol..

[81] Randerath K., Randerath E., Smith C.V., Chang J. (1996). Structural Origins of Bulky Oxidative DNA Adducts (Type II I-Compounds) as Deduced by Oxidation of Oligonucleotides of Known Sequence. Chem. Res. Toxicol..

[82] Barzilai A., Yamamoto K.I. (2004). DNA damage responses to oxidative stress. DNA Repair.

[83] Lips J., Kaina B. (2001). DNA double-strand breaks trigger apoptosis in p53-deficient fibroblasts. Carcinogenesis.

[84] Varga T., Aplan P.D. (2005). Chromosomal aberrations induced by double strand DNA breaks. DNA Repair.

[85] van Gent D.C., Hoeijmakers H.J., Kanaar R. (2001). Chromosomal stability and the DNA double-stranded break connection. Nat. Rev. Genet..

[86] Malins D.C., Polissar N.L., Gunselman S.J. (1996). Progression of human breast cancers to the metastatic state is linked to hydroxyl radical-induced DNA damage. Proc. Natl. Acad. Sci. USA.

[87] Li S.W., Lin T.-S., Minteer S., Burke W.J. (2001). 3,4-Dihydroxyphenylacetaldehyde and hydrogen peroxide generate a hydroxyl radical: Possible role in Parkinson’s disease pathogenesis. Mol. Brain Res..

[88] Lovell M.A., Markesbery W.R. (2007). Oxidative DNA damage in mild cognitive impairment and late-stage Alzheimer’s disease. Nucleic Acids Res..

[89] Valgimigli M., Merli E., Malagutti P., Soukhomovskaia O., Cicchitelli G., Antelli A., Canistro D., Francolini G., Macrì G., Mastrorilli F. (2004). Hydroxyl radical generation, levels of tumor necrosis factor-alpha, and progression to heart failure after acute myocardial infarction. J. Am. Coll. Cardiol..

[90] Talha M., Mir A.R., Habib S., Abidi M., Warsi M.S., Islam S., Moinuddin. (2021). Hydroxyl radical induced structural perturbations make insulin highly immunogenic and generate an auto-immune response in type 2 diabetes mellitus. Spectrochim. Acta A Mol. Biomol. Spectrosc..

[91] Wakatsuki A., Okatani Y., Izumiya C., Ikenoue N. (1999). Melatonin protects against ischemia and reperfusion-induced oxidative lipid and DNA damage in fetal rat brain. J. Pineal Res..

[92] Keshtkar Vanashi A., Ghasemzadeh H. (2022). Copper(II) containing chitosan hydrogel as a heterogeneous Fenton-like catalyst for production of hydroxyl radical: A quantitative study. Int. J. Biol. Macromol..

[93] Liu S., Zhao J., Zhang K., Yang L., Sun M., Yu H., Yan Y., Zhang Y., Wu L., Wang S. (2016). Dual-emissive fluorescence measurements of hydroxyl radicals using a coumarin-activated silica nanohybrid probe. Analyst.

[94] Louit G., Foley S., Cabillic J., Coffigny H., Taran F., Valleix A., Renault J.P., Pin S. (2005). The reaction of coumarin with the OH radical revisited: Hydroxylation product analysis determined by fluorescence and chromatography. Radiat. Phys. Chem..

[95] McCormick W.J., Rice C., McCrudden D., Skillen N., Robertson P.K.J. (2023). Enhanced Monitoring of Photocatalytic Reactive Oxygen Species: Using Electrochemistry for Rapid Sensing of Hydroxyl Radicals Formed during the Degradation of Coumarin. J. Phys. Chem. A.

[96] Manevich Y., Held K.D., Biaglow J.E. (1997). Coumarin-3-Carboxylic Acid as a Detector for Hydroxyl Radicals Generated Chemically and by Gamma Radiation. Radiat. Res..

[97] Cao D., Liu Z., Verwilst P., Koo S., Jangjili P., Kim J.S., Lin W. (2019). Coumarin-Based Small-Molecule Fluorescent Chemosensors. Chem. Rev..

[98] Xu Z., Chen Q., Zhang Y., Liang C. (2021). Coumarin-based derivatives with potential anti-HIV activity. Fitoterapia.

[99] King M., Kopelman R. (2003). Development of a hydroxyl radical ratiometric nanoprobe. Sens. Actuators B Chem..

[100] Soh N., Makihara K., Ariyoshi T., Seto D., Maki T., Nakajima H., Nakano K., Imato T. (2008). Phospholipid-linked Coumarin: A Fluorescent Probe for Sensing Hydroxyl Radicals in Lipid Membranes. Anal. Sci..

[101] Makrigiorgos G.M., Baranowska-kortylewicz J., Bump E., Sahu S.K., Berman R.M., Kassis A.I. (1993). A method for detection of hydroxyl radicals in the vicinity of biomolecules using radiation-induced fluorescence of coumarin. Int. J. Radiat. Biol..

[102] Makrigiorgos G.M., Folkard M., Huang C., Bump E., Baranowska-Kortylewicz J., Sahu S.K., Michael B.D., Kassis A.I. (1994). Quantification of Radiation-Induced Hydroxyl Radicals within Nucleohistones Using a Molecular Fluorescent Probe. Radiat. Res..

[103] Chakrabarti S., Makrigiorgos G.M., O’brien K., Bump E., Kassis A.I. (1996). Measurement of hydroxyl radicals catalyzed in the immediate vicinity of DNA by metal-bleomycin complexes. Free Radic. Biol. Med..

[104] Chakrabarti S., Mahmood A., Kassiss A.I., Bump E.A., Jones A.G., Makrigiorgos G.M. (1996). Generation of Hydroxyl Radicals by Nucleohistone-Bound Metal-Adriamycin Complexes. Free Radic. Res..

[105] Makrigiorgos G.M., Bump E., Huang C., Baranowska-Kortylewicz J., Kassis A.I. (1994). Accessibility of nucleic acid-complexed biomolecules to hydroxyl radicals correlates with their conformation: A fluorescence polarization spectroscopy study. Int. J. Radiat. Biol..

[106] Makrigiorgos G.M., Bump E., Huang C., Baranowska-Kortylewicz J., Kassis A.I. (1995). A fluorimetric method for the detection of copper-mediated hydroxyl free radicals in the immediate proximity of DNA. Free Radic. Biol. Med..

[107] Chakrabarti S. (1998). Continuous detection of radiation or metal generated hydroxyl radicals within core chromatin particles. Int. J. Radiat. Biol..

[108] Carter K.P., Young A.M., Palmer A.E. (2014). Fluorescent sensors for measuring metal ions in living systems. Chem. Rev..

[109] Kim I.J., Xu Y., Nam K.H. (2022). Metal-Induced Fluorescence Quenching of Photoconvertible Fluorescent Protein DendFP. Molecules.

[110] Singh A., Chen K., Adelstein S.J., Kassis A.I. (2007). Synthesis of Coumarin-Polyamine-Based Molecular Probe for the Detection of Hydroxyl Radicals Generated by Gamma Radiation. Radiat. Res..

[111] Singh A., Yang Y., Adelstein S.J., Kassis A.I. (2008). Synthesis and application of molecular probe for detection of hydroxyl radicals produced by Na ^125^I and γ-rays in aqueous solution. Int. J. Radiat. Biol..

[112] Tang V.J., Konigsfeld K.M., Aguilera J.A., Milligan J.R. (2012). DNA binding hydroxyl radical probes. Radiat. Phys. Chem..

[113] Leandri V., Gardner J.M., Jonsson M. (2019). Coumarin as a Quantitative Probe for Hydroxyl Radical Formation in Heterogeneous Photocatalysis. J. Phys. Chem. C.

[114] Leandri V., Gardner J.M., Jonsson M. (2019). Reply to “Comment on ‘Coumarin as a Quantitative Probe for Hydroxyl Radical Formation in Heterogeneous Photocatalysis’”. J. Phys. Chem. C.

[115] Nosaka Y., Nosaka A.Y. (2019). Comment on “Coumarin as a Quantitative Probe for Hydroxyl Radical Formation in Heterogeneous Photocatalysis”. J. Phys. Chem. C.

[116] Žerjav G., Albreht A., Vovk I., Pintar A. (2020). Revisiting terephthalic acid and coumarin as probes for photoluminescent determination of hydroxyl radical formation rate in heterogeneous photocatalysis. Appl. Catal. A Gen..

[117] Zhang J., Nosaka Y. (2013). Quantitative detection of OH radicals for investigating the reaction mechanism of various visible-light TiO_2_ photocatalysts in aqueous suspension. J. Phys. Chem. C.

[118] Newton G.L., Milligan J.R. (2006). Fluorescence detection of hydroxyl radicals. Radiat. Phys. Chem..

[119] Schuessel K., Frey C., Jourdan C., Keil U., Weber C.C., Müller-Spahn F., Müller W.E., Eckert A. (2006). Aging sensitizes toward ROS formation and lipid peroxidation in PS1M146L transgenic mice. Free Radic. Biol. Med..

[120] Valavanidis A., Vlachogianni T., Fiotakis C. (2009). 8-hydroxy-2′-deoxyguanosine (8-OHdG): A Critical Biomarker of Oxidative Stress and Carcinogenesis. J. Environ. Sci. Health C.

[121] Wallace D.C. (1997). Mitochondrial DNA in Aging and Disease. Sci. Am..

[122] Kim K.H., Park J.Y., Jung H.J., Kwon H.J. (2011). Identification and biological activities of a new antiangiogenic small molecule that suppresses mitochondrial reactive oxygen species. Biochem. Biophys. Res. Commun..

[123] Trachootham D., Alexandre J., Huang P. (2009). Targeting cancer cells by ROS-mediated mechanisms: A radical therapeutic approach?. Nat. Rev. Drug Discov..

[124] Zhou D., Huang H., Wang Y., Wang Y., Hu Z., Li X. (2019). A yellow-emissive carbon nanodot-based ratiometric fluorescent nanosensor for visualization of exogenous and endogenous hydroxyl radicals in the mitochondria of live cells. J. Mater. Chem. B.

[125] Huang X., Song J., Yung B.C., Huang X., Xiong Y., Chen X. (2018). Ratiometric optical nanoprobes enable accurate molecular detection and imaging. Chem. Soc. Rev..

[126] Thermo Fisher Scientific. https://www.thermofisher.com/order/catalog/product/M7512.

[127] Ma J., Kong X., Wang X., Xu Y., Zhao M., Xie H., Si W., Zhang Z. (2024). A dual-emission mitochondria targeting fluorescence probe for detecting hydroxyl radical and its generation induced by cellular activities. J. Mol. Liq..

[128] Chen J., Xu Y., Gao Y., Sun L., Meng X., Gu K., Zhang Y., Ning X. (2021). A mitochondria-specific fluorescent probe for rapidly assessing cell viability. Talanta.

[129] Goldman S.D.B., Funk R.S., Rajewski R.A., Krise J.P. (2009). Mechanisms of amine accumulation in, and egress from, lysosomes. Bioanalysis.

[130] Yuan G., Ding H., Sun H., Zhou L., Lin Q. (2019). A mitochondrion-targeting turn-on fluorescent probe detection of endogenous hydroxyl radicals in living cells and zebrafish. Sens. Actuators B Chem..

[131] Kubota C., Torii S., Hou N., Saito N., Yoshimoto Y., Imai H., Takeuchi T. (2010). Constitutive reactive oxygen species generation from autophagosome/lysosome in neuronal oxidative toxicity. J. Biol. Chem..

[132] He X., Li X., Tian W., Li C., Li P., Zhao J., Yang S., Li S. (2023). The role of redox-mediated lysosomal dysfunction and therapeutic strategies. Biomed. Pharmacother..

[133] Wang F., Gomez-Sintes R., Boya P. (2018). Lysosomal membrane permeabilization and cell death. Traffic.

[134] Ditaranto K., Tekirian T.L., Yang A.J. (2001). Lysosomal membrane damage in soluble Aβ-mediated cell death in Alzheimer’s disease. Neurobiol. Dis..

[135] Micsenyi M.C., Sikora J., Stephney G., Dobrenis K., Walkley S.U. (2013). Lysosomal membrane permeability stimulates protein aggregate formation in neurons of a lysosomal disease. J. Neurosci..

[136] Cash T.P., Alcalá S., Rico-Ferreira M.D.R., Hernández-Encinas E., García J., Albarrán M.I., Valle S., Muñoz J., Martínez-González S., Blanco-Aparicio C. (2020). Induction of lysosome membrane permeabilization as a therapeutic strategy to target pancreatic cancer stem cells. Cancers.

[137] LeGendre O., Breslin P.A.S., Foster D.A. (2015). (-)-Oleocanthal rapidly and selectively induces cancer cell death via lysosomal membrane permeabilization. Mol. Cell. Oncol..

[138] Benitez-Martin C., Guadix J.A., Pearson J.R., Najera F., Perez-Pomares J.M., Perez-Inestrosa E. (2019). A turn-on two-photon fluorescent probe for detecting lysosomal hydroxyl radicals in living cells. Sens. Actuators B Chem..

[139] Zhong L., Fu D., Xu J., Tan L., Wu H., Wang M. (2024). Rational design of a lysosome-targeted fluorescent probe for monitoring the generation of hydroxyl radicals in ferroptosis pathways. RSC Adv..

[140] Peng W., Wong Y.C., Krainc D. (2020). Mitochondria-lysosome contacts regulate mitochondrial Ca^2+^ dynamics via lysosomal TRPML1. Proc. Natl. Acad. Sci. USA.

[141] Peruzzo R., Costa R., Bachmann M., Leanza L., Szabò I. (2020). Mitochondrial metabolism, contact sites and cellular calcium signaling: Implications for tumorigenesis. Cancers.

[142] Burbulla L.F., Song P., Mazzulli J.R., Zampese E., Wong Y.C., Jeon S., Santos D.P., Blanz J., Obermaier C.D., Strojny C. (2017). Dopamine oxidation mediates mitochondrial and lysosomal dysfunction in Parkinson’s disease. Science.

[143] Cisneros J., Belton T.B., Shum G.C., Molakal C.G., Wong Y.C. (2022). Mitochondria-lysosome contact site dynamics and misregulation in neurodegenerative diseases. Trends Neurosci..

[144] Ma J., Zhao M., Kong X., Xie H., Li H., Jiao Z., Zhang Z. (2024). An innovative dual-organelle targeting NIR fluorescence probe for detecting hydroxyl radicals in biosystem and inflammation models. Bioorganic Chem..

[145] Wang L.L., Mai Y.Z., Zheng M.H., Wu X., Jin J.Y. (2022). Fluorescent probe disclosing hydroxyl radical generation in mitochondria and nucleoli of cells during ferroptosis. Sens. Actuators B Chem..

[146] Darzynkiewicz Z., Kapuscinski J., Carter S.P., Schmid F.A., Melamed M.R. (1986). Cytostatic and Cytotoxic Properties of Pyronin Y: Relation to Mitochondrial Localization of the Dye and Its Interaction with RNA. Cancer Res..

[147] Wang B., Wang Y., Zhang J., Hu C., Jiang J., Li Y., Peng Z.Y. (2023). ROS-induced lipid peroxidation modulates cell death outcome: Mechanisms behind apoptosis, autophagy, and ferroptosis. Arch. Toxicol..

[148] Sánchez-Pérez Y., Carrasco-Legleu C., García-Cuellar C., Pérez-Carreón J., Hernández-García S., Salcido-Neyoy M., Alemán-Lazarini L., Villa-Treviño S. (2005). Oxidative stress in carcinogenesis. Correlation between lipid peroxidation and induction of preneoplastic lesions in rat hepatocarcinogenesis. Cancer Lett..

[149] Suryawanshi N.P., Bhutey A.K., Nagdeote A.N., Jadhav A.A., Manoorkar G.S. (2006). Study of lipid peroxide and lipid profile in diabetes mellitus. Indian. J. Clin. Biochem..

[150] Toborek M., Kopieczna-Grzebieniak E., Drbzdz M., Wieczorekb M. (1995). Increased lipid peroxidation as a mechanism of methionine-induced atherosclerosis in rabbits. Atherosclerosis.

[151] Nawara K., Waluk J. (2017). Improved Method of Fluorescence Quantum Yield Determination. Anal. Chem..

[152] Patsenker L., Tatarets A., Kolosova O., Obukhova O., Povrozin Y., Fedyunyayeva I., Obukhova I., Terpetschnig E. (2008). Fluorescent Probes and Labels for Biomedical Applications. Ann. N.Y. Acad. Sci..

[153] Ross M.F., Da Ros T., Blaikie F.H., Prime T.A., Porteous C.M., Severina I.I., Skulachev V.P., Kjaergaard H.G., Smith R.J., Murphy M.P. (2006). Accumulation of lipophilic dications by mitochondria and cells. Biochem. J..

[154] Sulkin M.S., Boukens B.J., Tetlow M., Gutbrod S.R., Siong Ng F., Efimov I.R., Efimov I.R. (2014). Mitochondrial depolarization and electrophysiological changes during ischemia in the rabbit and human heart. Am. J. Physiol. Heart Circ. Physiol..

[155] Lyamzaev K.G., Tokarchuk A.V., Panteleeva A.A., Mulkidjanian A.Y., Skulachev V.P., Chernyak B.V. (2018). Induction of autophagy by depolarization of mitochondria. Autophagy.

[156] Jean S.R., Ahmed M., Lei E.K., Wisnovsky S.P., Kelley S.O. (2016). Peptide-Mediated Delivery of Chemical Probes and Therapeutics to Mitochondria. Acc. Chem. Res..

[157] Kazmi F., Hensley T., Pope C., Funk R.S., Loewen G.J., Buckley D.B., Parkinson A. (2013). Lysosomal sequestration (trapping) of lipophilic amine (cationic amphiphilic) drugs in immortalized human hepatocytes (Fa_2_N-4 cells). Drug Metab. Dispos..

[158] Liu Y.-Z., Zhang H., Zhou D.-H., Liu Y.-H., Ran X.-Y., Xiang F.-F., Zhang L.-N., Chen Y.-J., Yu X.-Q., Li K. (2023). Migration from Lysosome to Nucleus: Monitoring Lysosomal Alkalization-Related Biological Processes with an Aminofluorene-Based Probe. Anal. Chem..

[159] Zhang T., Hong X.-Q., Zhi H.-T., Jinhui H., Chen W.-H. (2022). Synthesis and mechanism of biological action of morpholinyl-bearing arylsquaramides as small-molecule lysosomal pH modulators. RSC Adv..

[160] Wolfbeis O.S. (2015). An overview of nanoparticles commonly used in fluorescent bioimaging. Chem. Soc. Rev..

[161] Han X., Xu K., Taratula O., Farsad K. (2019). Applications of nanoparticles in biomedical imaging. Nanoscale.

[162] Guo Z., Park S., Yoon J., Shin I. (2014). Recent progress in the development of near-infrared fluorescent probes for bioimaging applications. Chem. Soc. Rev..

[163] Galievsky V.A., Stasheuski A.S., Krylov S.N. (2017). Improvement of LOD in fluorescence detection with spectrally nonuniform background by optimization of emission filtering. Anal. Chem..

[164] Wittenberg N.J., Haynes C.L. (2009). Using nanoparticles to push the limits of detection. Wiley Interdiscip. Rev. Nanomed. Nanobiotechnol..

[165] Lee J., Giordano S., Zhang J. (2012). Autophagy, mitochondria and oxidative stress: Cross-talk and redox signalling. Biochem. J..

[166] Deus C.M., Yambire K.F., Oliveira P.J., Raimundo N. (2020). Mitochondria–lysosome crosstalk: From physiology to neurodegeneration. Trends Mol. Med..

[167] Sheu S.-S., Nauduri D., Anders M.W. (2006). Targeting antioxidants to mitochondria: A new therapeutic direction. Biochim. Biophys. Acta Mol. Basis Dis..

[168] Asayama S., Kawamura E., Nagaoka S., Kawakami H. (2006). Design of Manganese Porphyrin Modified with Mitochondrial Signal Peptide for a New Antioxidant. Mol. Pharm..

[169] He J., Ma Y., Niu X., Pei J., Yan R., Xu F., Ma J., Ma X., Jia S., Ma W. (2024). Silver nanoparticles induce endothelial cytotoxicity through ROS-mediated mitochondria-lysosome damage and autophagy perturbation: The protective role of N-acetylcysteine. Toxicology.

